# Quantifying ADC bystander payload penetration with cellular resolution using pharmacodynamic mapping

**DOI:** 10.1016/j.neo.2020.12.001

**Published:** 2020-12-29

**Authors:** Eshita Khera, Cornelius Cilliers, Michael D. Smith, Michelle L. Ganno, Katharine C. Lai, Thomas A. Keating, Anna Kopp, Ian Nessler, Adnan O. Abu-Yousif, Greg M. Thurber

**Affiliations:** a Department of Chemical Engineering, University of Michigan, Ann Arbor, MI, USA; b Department of Biomedical Engineering, University of Michigan, Ann Arbor, MI, USA; c Millennium Pharmaceuticals Inc., Cambridge, MA, USA; d Immunogen Inc., Waltham, MA, USA

**Keywords:** ADC bystander effect, Pharmacodynamic marker, Primary human tumor xenograft, Tumor-associated macrophages, Tumor spheroids, ADCs, antibody drug conjugates, BSA, bovine serum albumin, GCC, guanylyl-cyclase C, MTD, maximum tolerated dose, PBS, phosphate-buffered saline, PHTX, primary human tumor xenograft, SBE, spatial bystander effects, TAM, tumor-associated macrophages

## Abstract

With the recent approval of 3 new antibody drug conjugates (ADCs) for solid tumors, this class of drugs is gaining momentum for the targeted treatment of cancer. Despite significant investment, there are still fundamental issues that are incompletely understood. Three of the recently approved ADCs contain payloads exhibiting bystander effects, where the payload can diffuse out of a targeted cell into adjacent cells. These effects are often studied using a mosaic of antigen positive and negative cells. However, the distance these payloads can diffuse in tumor tissue while maintaining a lethal concentration is unclear. Computational studies suggest bystander effects partially compensate for ADC heterogeneity in tumors in addition to targeting antigen negative cells. However, this type of study is challenging to conduct experimentally due to the low concentrations of extremely potent payloads. In this work, we use a series of 3-dimensional cell culture and primary human tumor xenograft studies to directly track fluorescently labeled ADCs and indirectly follow the payload via an established pharmacodynamic marker (γH2A. X). Using TAK-164, an anti-GCC ADC undergoing clinical evaluation, we show that the lipophilic DNA-alkylating payload, DGN549, penetrates beyond the cell targeted layer in GCC-positive tumor spheroids and primary human tumor xenograft models. The penetration distance is similar to model predictions, where the lipophilicity results in moderate tissue penetration, thereby balancing improved tissue penetration with sufficient cellular uptake to avoid significant washout. These results aid in mechanistic understanding of the interplay between antigen heterogeneity, bystander effects, and heterogeneous delivery of ADCs in the tumor microenvironment to design clinically effective therapeutics.

## Introduction

Antibody-drug conjugates (ADC) have witnessed expansive growth in the last decade with U.S. Food and Drug Administration (FDA) approval of 9 ADCs and several more in clinical trials. ADCs consist of 3 main components – (1) An antibody/protein backbone with antigen-specific targeting capabilities, (2) a cytotoxic small molecule payload, and (3) a chemical/peptide linker that stably conjugates the antibody to the payload. These drugs have evolved considerably since the first generation introduced nearly 4 decades ago, driven by biophysical improvements that have enabled the exploration of several antibody backbones, linker types, conjugation chemistries, and payloads [1]. The selection of the ADC payload remains largely empirical despite being the most prominently diversified component in next-generation ADCs [2] that employs moderate (e.g., SN-38), high (maytansinoids, auristatins, etc.), and ultra-high (DNA-interacting) potency payloads. Clinical translation of moderate to high potency payloads (nM IC_50_) have resulted in several approvals, but ultra-high payload potency (pM IC_50_) has proven to be a double-edged sword, inversely scaling with the *in vivo* maximum tolerated dose (MTD) of the ADC. Low MTD can result in heterogenous perivascular distribution, which is a tremendous challenge for ADCs targeting solid tumors, as seen by the FDA-approval of just 4 solid tumor ADCs in the last decade. Heterogeneous antigen expression is another common clinical feature of solid tumors [3], and while targeted ADCs can efficiently kill Ag-positive (Ag+) cells, Ag-negative (Ag-) cells remain unexposed to the payload and survive. Both heterogeneous ADC distribution and antigen expression can contribute to poor clinical efficacy, but both mechanisms can be compensated by bystander killing, where the payload can diffuse from ADC-targeted to untargeted cells.

ADC payloads are broadly categorized as “nonbystander” or “bystander,” usually based on *in vitro* Ag+/Ag- co-culture assays [4] which often cannot describe the precise bystander penetration distance. Quantification of the distance a bystander payload can penetrate before being sufficiently diluted in tissue to noncytotoxic concentrations is crucial for designing more clinically efficient ADCs, particularly for clinical tumors that do not always have Ag+ and Ag- cells closely interspersed, or where untargeted regions may lie far beyond the “binding site barrier” [5]. However, direct spatiotemporal tracking of bystander payloads is challenging. Fluorescence is often used as a proxy to track molecules, and fluorophore-tagging of antibodies can be achieved without significantly altering their physicochemical and pharmacokinetic properties [6]. However, for small molecule payloads, organic fluorophores are the same size as the drug itself, considerably altering their pharmacokinetic behavior [7,8]. Conjugating the payload to an appropriate radiolabel is a viable option but greatly depends on the sensitivity and spatial resolution of signal detection. For example, the less lipophilic MMAE can penetrate a couple hundred microns beyond the intact ADC in the tumor [9], which can be detected with sufficient sensitivity and resolution via dual-isotope radioimaging [10]. In contrast, more lipophilic DNA-interacting bystander payloads are predicted to diffuse more slowly in the tumor extracellular space before irreversibly partitioning into cells, with gradients occurring over shorter distances (∼ several tens of microns). Tracking distribution of such ultrapotent payloads is nontrivial because – (1) radiolabeled ultrapotent payloads have tolerability limitations, resulting in lower absolute concentrations compared to MMAE, and (2) the predicted penetration distances are shorter than MMAE, requiring high sensitivity and cellular-level resolution not achievable by radioimaging. However, this same ultra-high potency can manifest as strong cellular pharmacodynamic effects even at low payload exposure, enabling the deployment of pharmacodynamic markers as tools for monitoring tumor penetration of highly lipophilic ultra-potent payloads. In particular, DNA-interacting payloads mediate cytotoxicity by generating double-stranded DNA breaks (DSBs) which triggers the phosphorylation of Ser^139^ of the H2A.X histone protein (γH2A.X) [[11], [12], [13]]. This in turn can be detected sensitively and with cellular resolution via immunofluorescent staining and is a well-established tool in the field of molecular cancer research.

In this study, we employ TAK-164, a guanylyl-cyclase C (GCC)-targeted ADC currently under clinical evaluation for colorectal cancer and GI malignancy indications carrying DGN549, a potent DNA-alkylating payload with reported bystander effects [14], to pharmacodynamically map bystander payload tissue penetration. Through immunofluorescence imaging of a series of 3D *in vitro* cell culture and primary human tumor xenografts (PHTX) tumor studies, we directly tracked fluorescently labeled TAK-164 and DGN549 toxicity via γH2A.X signal. We used this system to experimentally evaluate the distribution of a DNA-damage payload from a class of payloads with predicted “ideal” physicochemical properties [9]. The observed penetration distance was compared to model predictions, highlighting how the lipophilicity of DGN549 impacts tissue penetration. These results represent a critical step in mechanistic understanding of the interplay between antigen heterogeneity, bystander effects, and heterogeneous delivery of ADCs in the tumor microenvironment, which can aid in designing more effective therapeutics.

## Materials and methods

### Computational bystander model

Computational simulations of ADC and payload distribution were performed using a previously published Krogh cylinder model [9], adapted to spherical geometry for tumor spheroid simulations (Supplementary Figure 1). ADC kinetic parameters were empirically extracted from independently performed *in vitro* experiments and payload kinetic parameters were estimated using literature data and previously published methodology [9] (Supplementary Table 1). Differential equations and model parameters have been detailed in the Supplementary Information. To help visualize the data, cells that accumulate payload beyond a “therapeutic threshold” of ∼30 nM were expected to show consistent γH2A.X signal. The therapeutic threshold for DGN549 was estimated based on dose-dependent analysis of experimental γH2A.X signal, measured cellular viability, and estimated cellular payload concentrations (Supplementary Figure 2). This model framework has previously been developed for *a priori* prediction of bystander effects; however, parameters were updated to account for a nonlinear antibody internalization rate and measured free payload tissue penetration in spheroids (Supplementary Figure 3).

### Antibody constructs and fluorescent labeling

As described previously, the recombinant anti-GCC antibody 5F9 was produced [15] and conjugated to sulfonated-DGN549 to generate TAK-164 [16]. Both wild type (5F9, TAK-164-WT) and Fc-mutant (5F9-FcM, TAK-164-FcM) constructs were provided by Millennium Pharmaceuticals Inc. (wholly owned subsidiary of Takeda Pharmaceutical Co. Ltd.) Fluorophores (AlexaFluor 488, 555, 647, 680, and 750) were purchased from ThermoFisher Scientific. TAK-164 was first buffer exchanged into phosphate-buffered saline (PBS) using Zeba Spin Desalting Column (ThermoFisher Scientific). Lysine residues on 5F9 and desalted TAK-164 were conjugated to AlexaFluor647 (AF647) or AlexaFluor680 (AF680) via NHS-amine chemistry as described previously [6]. Concentration and degree-of-labeling (DoL; number of dyes per antibody) typically ranged from 10 to 20 µM and 0.7 to 1.2 DoL. Purified fluorescent antibody/ADC was analyzed via denaturing SDS-PAGE to ensure all free dye was removed. Antibodies for immunohistochemistry were fluorescently labeled in-house via Lysine-NHS chemistry, except for fluorescein-isothiocyanate (FITC) goat antirabbit secondary antibody (Novus Biologicals).

### Cell culture

Transfected HEK293-GCC cells were generated at Millennium Pharmaceuticals Inc. Cells were cultured at 37°C and 5% CO_2_ in Dulbecco's Modified Eagle's Medium supplemented with 10% fetal bovine serum and 10 µg/mL blasticidin (Invitrogen). Cells were passaged 2 to 3 times per week (∼80%–90% confluency) and checked for consistent GCC expression prior to performing assays. RAW 264.7 cells were purchased from ATCC (Manassas, VA) and cultured at 37°C and 5% CO_2_ in Dulbecco's Modified Eagle's Medium supplemented with 10% fetal bovine serum, 50 U/mL penicillin, and 50 µg/mL streptomycin.

### Tumor xenograft studies in mice

All animal studies were approved and performed in accordance with the Institutional Animal Care and Use Committee of the University of Michigan and Association for Assessment and Accreditation of Laboratory Animal Care International guidelines. Primary human tumor xenografts (PHTX 09C, PHTX 11C, PHTX 17C) tumors [15] were initiated in 6 to 7-wk-old female CB17 SCID mice, and subsequently propagated in 8-wk-old female Fox1^nu/nu^ nude mice under ABSL2 conditions. PHTX tumors were propagated by implanting viable tumor pieces cut into 2 × 2 × 2 mm^3^ fragments subcutaneously in the right flank of mice. HEK293-GCC tumor xenografts were propagated by inoculating ∼5 × 10^6^ cells in Matrigel subcutaneously in the right flank. Tumor volume was monitored using calipers 3 times a wk and calculated as 0.5 × length × width^2^. Tumor distribution studies were performed when tumors reached 100 to 250 mm^3^. To evaluate intratumoral distribution of TAK-164 in PHTX tumors, mice with appropriate size tumors were injected intravenously with 0.4 mg/kg or 1.5 mg/kg of AF647-TAK-164 (PHTX models) or 0.75 mg/kg AF647-TAK-164 (HEK293-GCC tumors). Mice were euthanized and tumors harvested 24 h or 72 h postinjection. Ten min prior to euthanasia, the mice were administered 15 mg/kg Hoechst-33342 intravenously to mark functional blood vessels. Resected tumors were either digested using a human tumor dissociation kit (Miltenyi Biotech) or flash frozen in OCT using isopentane chilled on dry ice for immunohistochemistry.

### γH2A.X pharmacodynamic measurement

For *in vitro* measurement of payload-induced phosphorylation of H2A.X, 7 × 10^4^ HEK293-GCC cells were plated overnight and incubated with fluorescent TAK-164 (concentration course for 48 h with daily media replacement or time course at 50 nM concentration). Postincubation, cells were washed in PBS, fixed with 4% formaldehyde (BD Cytofix), and permeabilized/blocked in 0.5% bovine serum albumin (BSA) in 1X perm buffer (BD Cytoperm) in PBS (0.5% BSA/perm) for 15 min each at room temperature. The Phospho-Histone H2A.X (Ser139) (20E3) rabbit primary antibody (Cell Signaling Technologies) was diluted to 0.15 µg/mL in 0.5% BSA/perm and incubated with permeabilized cells for 30 min in the dark at room temperature. Cells were washed twice for 5 min each with 0.5% BSA/perm. Secondary antibody incubation was performed with AF555-labeled goat antirabbit antibody diluted to 2.5 µg/mL in 0.5% BSA/perm for 30 min in the dark at room temperature. Cells were washed twice for 5 min each with 0.5% BSA/perm, followed by 2-min incubation with 0.5 mg/mL Hoechst 33342 in PBS. *In vivo* tumor sections were prepared similarly. Flow cytometry cells for γH2A.X measurement were prepared similarly, except in suspension and with FITC-goat antirabbit secondary antibody (also diluted to 2.5 µg/mL).

### Macrophage uptake

To determine if 5F9/TAK-164 uptake in macrophages is GCC-mediated or Fc-mediated, confluent RAW cells were harvested and labeled with 50 nM of AF647-5F9 for 30 min on ice with and without preblocking with 1µM “cold” (i.e., nonfluorescent) 5F9 or trastuzumab. Additionally, RAW cells were incubated with 50 nM AF647-F(ab’)_2_ (lacking the Fc domain to bind Fc-receptors), with and without preblocking with cold 5F9 or trastuzumab. Labeled cells were washed twice with PBS and analyzed by flow cytometry. To evaluate the contribution of tumor-associated macrophage mediated bystander effects, mice bearing PHTX 11C tumors were administered one of the following 4 ADC regimens intravenously – (1) 0.4 mg/kg of fluorescent wild-type TAK-164, (2) 0.4 mg/kg of fluorescent TAK-164 with a mutant Fc-component (TAK-164-FcM containing ELLG (pos. 116–120) to PVA [17]) to reduce binding to FcγR, (3) preblocking with 6mg/kg cold 5F9-FcM for 24 h followed by 0.4 m/kg fluorescent TAK-164-WT, and (4) preblocking with 6 mg/kg cold 5F9-FcM for 24 h followed by 0.4 m/kg fluorescent TAK-164-FcM. Mice were euthanized 72 h after fluorescent TAK-164 injection, with each being injected intravenously with 15 mg/kg Hoechst 33342 10 min prior to euthanasia. Resected tumors were flash frozen in OCT and processed for histology.

### Flow cytometry

Tumor digests were filtered through a 40 µm filter to remove tumor clumps and form a single-cell suspension. Cells were analyzed via flow cytometry, either directly (to measure signal from injected ADC) or after ex-vivo labeling for 30 min on ice with (1) AF647-5F9 (50nM), (2) primary γH2A.X antibody, and secondary FITC-goat antirabbit antibody for γH2A.X staining, or (3) secondary FITC goat antirabbit antibody only for nonspecific sticking contribution to γH2A.X signal, followed by 2x washes with PBS. Data were analyzed using FlowJo software. Fraction receptors targeted was calculated as ratio of median fluorescence from intravenously administered AF647-TAK-164 to median fluorescence of cells from same tumor labeled ex-vivo with AF647-5F9 (similar DoL).

### Immunohistochemistry

Tumors frozen in OCT were sectioned on a cryostat (16 µm slices) and processed for histology. For each tumor, 2 identical slices were processed side-by-side for (A) pharmacokinetic and (B) pharmacodynamic staining. Slide A was incubated with AF555-anti-mouse CD31 antibody (BioLegend, 102402), AF488-anti-Mac3 antibody (BD Biosciences, 553322) and AF750-5F9 in 0.5% BSA/PBS for 30 min in the dark at room temperature, then washed twice for 5 min with PBS. Microscopy was performed using an upright Olympus FV1200 confocal microscope using a 20× objective and 405 nm (injected Hoechst 33342; active blood vessels), 488 nm (Mac3; macrophages), 543 nm (CD31; all blood vessels), 635 nm (injected TAK-164), and 748 nm (AF75-5F9; all accessible GCC) lasers. Slide B was incubated with primary γH2A.X antibody for 30 min followed by AF555-goat antirabbit (GAR) secondary antibody for 30 min, followed by 0.5 mg/mL Hoechst 33342, with 2x PBS washes between incubations. Imaging was performed similar to slide A, using only 405 nm (ex vivo Hoechst 33342; all nuclei), 543 nm (γH2A.X), and 635 nm (injected TAK-164) lasers. High resolution images of continuous sections were captured through multiarea imaging and stitched together on the Olympus software to form a single high-resolution image of each tumor slice. Image processing was performed using ImageJ image analysis software.

### Tumor spheroid experiments

HEK293-GCC spheroids were cultured using custom 384-well plates described previously [18]. Briefly, spheroids were grown for 7 d with media changes until they reached 500 to 600 µm in diameter [19]. To evaluate TAK-164 distribution in spheroids, hanging drops were “pulsed” in 30 nM fluorescent TAK-164 or 5F9 for 9 h or 16 h, after which the drug was washed away, and spheroids “chased” in media up to 54 h to allow for DGN549-mediated DNA damage. Spheroids incubated continuously with fluorescent 5F9/TAK-164 for 54 h were used as negative/positive control respectively for γH2A.X staining. Final spheroid incubation conditions were determined based on time course spheroid incubations with fluorescent 5F9 (Supplementary Figure 1C) to capture increasing bystander/γH2A.X response. Spheroids were processed similar to tumors for histology and images analyzed using ImageJ and MATLAB.

## Results

### γH2A.X pharmacodynamic marker detects DNA-interacting bystander payload

**Fig. 1:**
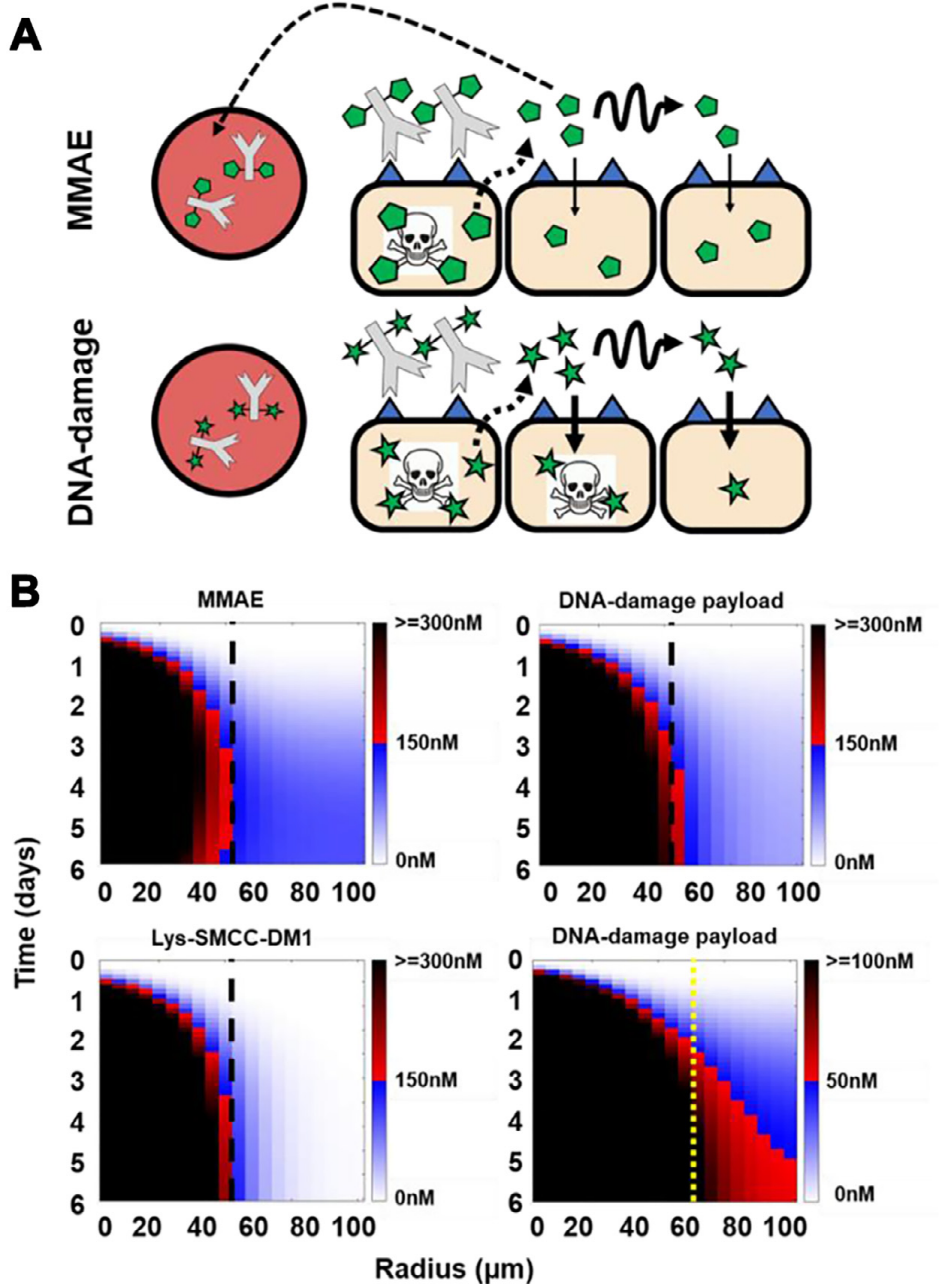
Tracking distribution of bystander payloads. (A) Schematic highlighting the varying bystander penetration and killing of MMAE (monomethyl auristatin E) and irreversible DNA-interacting/damage payloads (e.g., pyrrolobenzodiazepine). (B) Krogh cylinder model simulating payload distribution shows faster diffusion of MMAE (logD = 2.01) which binds quickly but reversibly to microtubules compared to more potent and lipophilic DNA-interacting payloads like PBD (logD = 4.12) which bind slowly and irreversibly to DNA. The faster diffusion of MMAE results in more homogeneous bystander penetration, and higher tolerated doses result in higher concentrations through the tumor compared to DNA-interacting payloads, limiting the use of radioimaging for the latter. The relatively slower cell uptake results in more MMAE washout, reducing pharmacodynamic bystander killing efficiency compared to DNA-interacting payloads. *Black dashed line indicates cytotoxic penetration front of nonbystander payload Lys-SMCC-DM1 (bottom left)*. Furthermore, since DNA-interacting payloads are typically 10- to 100-fold more potent than MMAE, accumulation at even 3-fold lower concentrations (scale bar reduced 3-fold in bottom right) can be sufficient to induce a pharmacodynamic effect. *Yellow dashed line indicates cytotoxic penetration front of nonbystander payload Lys-SMCC-DM1 at the lower threshold (0-50-100nM), to show the better efficacy of DNA-damage payload at distances type="Other">60 microns is a result of bystander killing. Boundary between red-black gradient (dead cells) and blue-white gradient (viable cells) represents the cytotoxic threshold for the simulations*

Predictive Krogh cylinder modeling shows that both MMAE and DNA-interacting payloads both behave as bystander payloads, but the latter only diffuse several cell layers in the tumor before internalizing into cells. This contrasts with the less lipophilic MMAE, which diffuses much faster than its cellular internalization rate, resulting in overall greater extracellular penetration but less accumulation in cells and more washout from the tumor (Figure 1A). Dimensional analysis predicts that the DNA-interacting payload behaves as an “ideal” bystander payload [9] since considerably more ADC-untargeted cells accumulate a lethal dose of the payload, while a faster diffusion rate causes more payload to wash out of the tumor (Figure 1B). Unlike for MMAE [10], radioimaging did not provide sufficient sensitivity, resolution, or tolerability to verify the distribution of the slower diffusing DNA-interacting payload on TAK-164 (data not shown). Instead, we employed a well-established γH2A.X pharmacodynamic marker as a proxy to detect DNA-damage by staining for the formation of H2A.X Ser^139^ phosphorylation foci in the nucleus in response to payload-induced dsDNA breaks (Supplementary Figure 4). Time-dependent incubation of HEK293-GCC cells *in vitro* with TAK-164 showed increasing γH2A.X signal after 24 h, consistent with the time frame required for antibody internalization, payload release, and DNA damage (Supplementary Figure 5). Incubation of HEK293-GCC monolayer cells with increasing concentrations of fluorescently labeled TAK-164 (*green*) showed a dose-dependent γH2A.X response, evidenced by the formation of increasing number of nuclear-specific foci (*red*) with increasing dose (Supplementary Figure 4).

### In vivo model for mapping bystander payload penetration

DNA-interacting molecules are ultra-potent payloads with a picomolar IC_50_, which results in relatively low maximum tolerated doses, making such ADCs most suitable for tumors with low to moderate antigen expression [20]. The lower antigen expression can allow deeper tissue penetration of the ADC, while the strong potency can offset the reduced intracellular payload accumulation to mediate more efficient tumor killing. TAK-164 is a highly potent ADC undergoing clinical evaluation that consists of the DNA-alkylating payload DGN549 and an antibody component (5F9) with strong affinity to GCC (guanylyl cyclase C) *in vitro* (Supplementary Figure 6). Previous analyses of patient colorectal tumors (CRC) shows > 95% of CRCs express GCC, which is consistent throughout cancer progression, while the level of expression is moderate [21], making this a promising system to explore bystander payload penetration of DNA-interacting payloads.

**Fig. 2:**
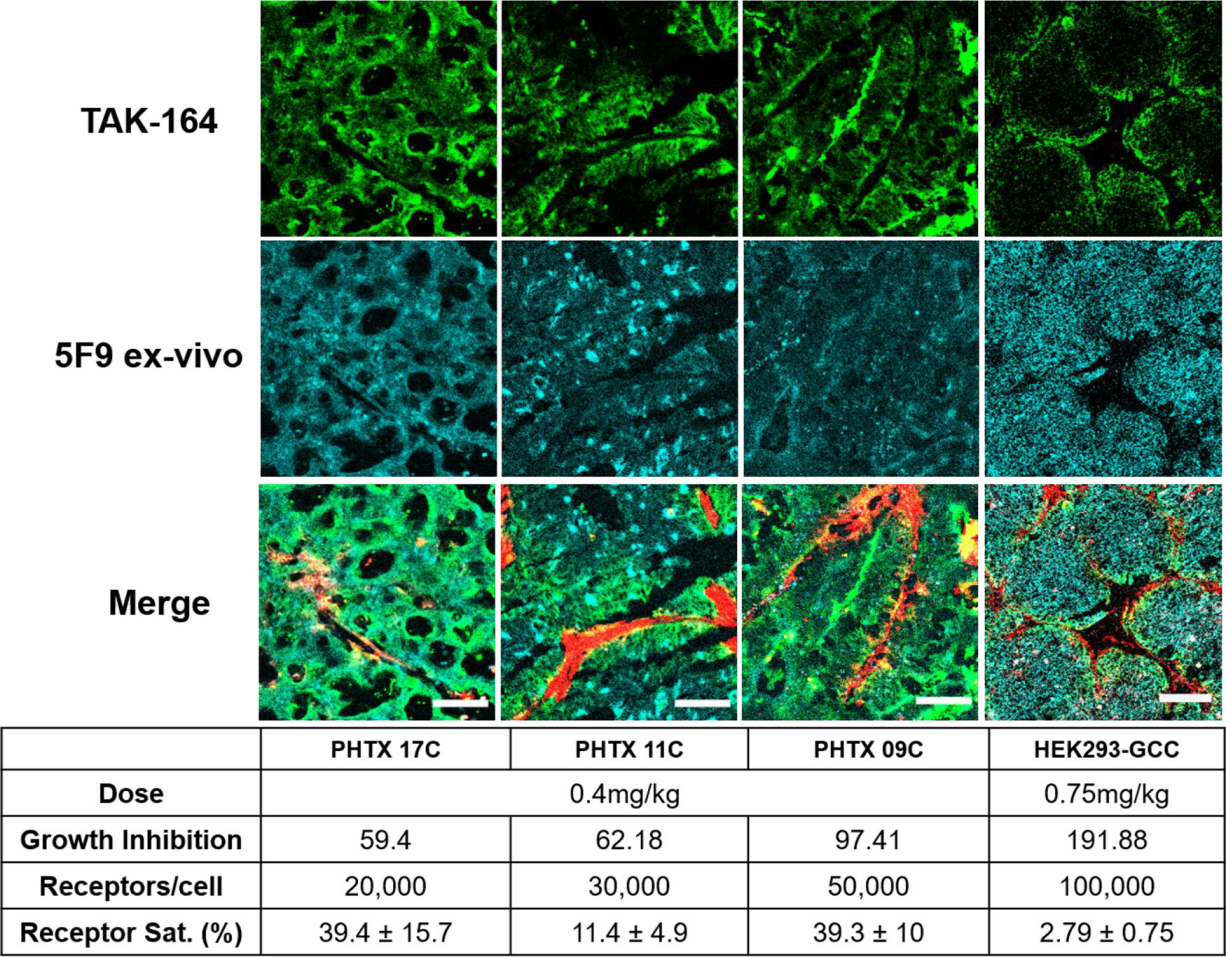
Selection of tumor model(s) to study bystander payload penetration *in vitro* and *in vivo*. Four tumor models - 3 primary human tumor xenografts (PHTX 17C, 11C, 09C) and one transfected cell line (HEK293-GCC), each with varying receptor expression and auristatin sensitivity - were evaluated to identify tumor model(s) to study bystander payload penetration. PHTX 17C and 09C showed relatively more homogeneous TAK-164 distribution, with 40% of GCC receptors targeted with 0.4 mg/kg of TAK-164. In contrast, PHTX 11C and HEK293-GCC xenograft tumors showed heterogeneous TAK-164 distribution, with a low percent of receptors targeted by the injected dose of TAK-164, giving the best physical separation between ADC-targeted and untargeted cells. An auristatin-refractory (15) PHTX 11C model was selected for studying DGN549 tumor penetration *in vivo*, while HEK293-GCC (which can be grown into spheroids) model was selected for *in vitro* studies. Scale bar = 100 μm. Growth inhibition data from Abu-Yousif. A.O. *et al*., Mol. Can Ther. (In press, 2020). *green = AF647-TAK-164, cyan = ex-vivo AF750-5F9, red = Mac3-AF488 (stroma surrounding blood vessels)*

One challenge in studying the tissue penetration of bystander payloads and associated bystander effects *in vivo* is distinguishing payload delivered via direct ADC uptake versus payload delivered via bystander diffusion. To circumvent this problem, we used tumor xenograft models exhibiting heterogeneous ADC delivery, thereby allowing physical separation of directly targeted cells versus untargeted cells (that only exhibit bystander killing). We evaluated TAK-164 distribution in 4 tumor models: HEK293-GCC xenografts and 3 Primary Human colon Tumor Xenografts, PHTX 09C, PHTX 11C, and PHTX 17C, all of which have been characterized previously [15]. Immunohistochemistry showed each model had moderate GCC expression [15], which was confirmed by quantitative flow cytometry analysis (between 10^4^and 10^5^ receptors/cell) (Supplementary Figure 7). Flow cytometry analysis and immunofluorescence imaging of tumors treated intravenously with 0.4 mg/kg (PHTX) or 0.75 mg/kg (HEK293-GCC) fluorescent TAK-164 showed distinct patterns of receptor targeting across all 4 models (Figure 2). PHTX 17C and PHTX 09C showed the highest fraction of receptors targeted (∼40%) by 0.4 mg/kg TAK-164, followed by PHTX 11C (11%). HEK293-GCC tumors showed the lowest fraction of receptors targeted, even with double the dose of 0.75 mg/kg TAK-164 (∼3%). Note, these numbers represent the median receptors targeted, an important consideration as HEK293-GCC tumors show homogeneous antigen expression while the PHTX tumors show varying localization and expression patterns (e.g., acinar pockets, Figure 2). Immunofluorescence imaging of tumor cross-sections revealed near homogeneous intratumoral distribution of TAK-164 in PHTX 17C and PHTX 09C, but PHTX 11C and HEK293-GCC showed distinctly heterogeneous distribution. Interestingly, PHTX 11C and PHTX 17C have similar receptor expression but varying tissue penetration, indicating that receptor expression alone is not always a predictor of ADC distribution patterns. Additionally, despite only 3% receptors targeted and perivascular distribution, HEK293-GCC tumors show complete regression at 0.75 mg/kg [16], suggesting that DGN549, a known bystander payload, might be capable of penetrating considerable distance in the tumor while still accumulating to sufficient concentrations to mediate cytotoxicity as predicted by our previous mechanistic model [9]. Based on the heterogeneous distribution (resulting in physical separation between ADC-targeted and ADC-untargeted cells), PHTX 11C and HEK293-GCC were selected for studying bystander effects. HEK293-GCC is a cell-based system that can be used to derive tumor spheroids for 3D tissue culture studies while the PHTX 11C provides a more complex tumor microenvironment to better replicate a clinical setting.

### Directly observable, in vivo cellular-resolution evidence of spatial bystander effects

**Fig. 3:**
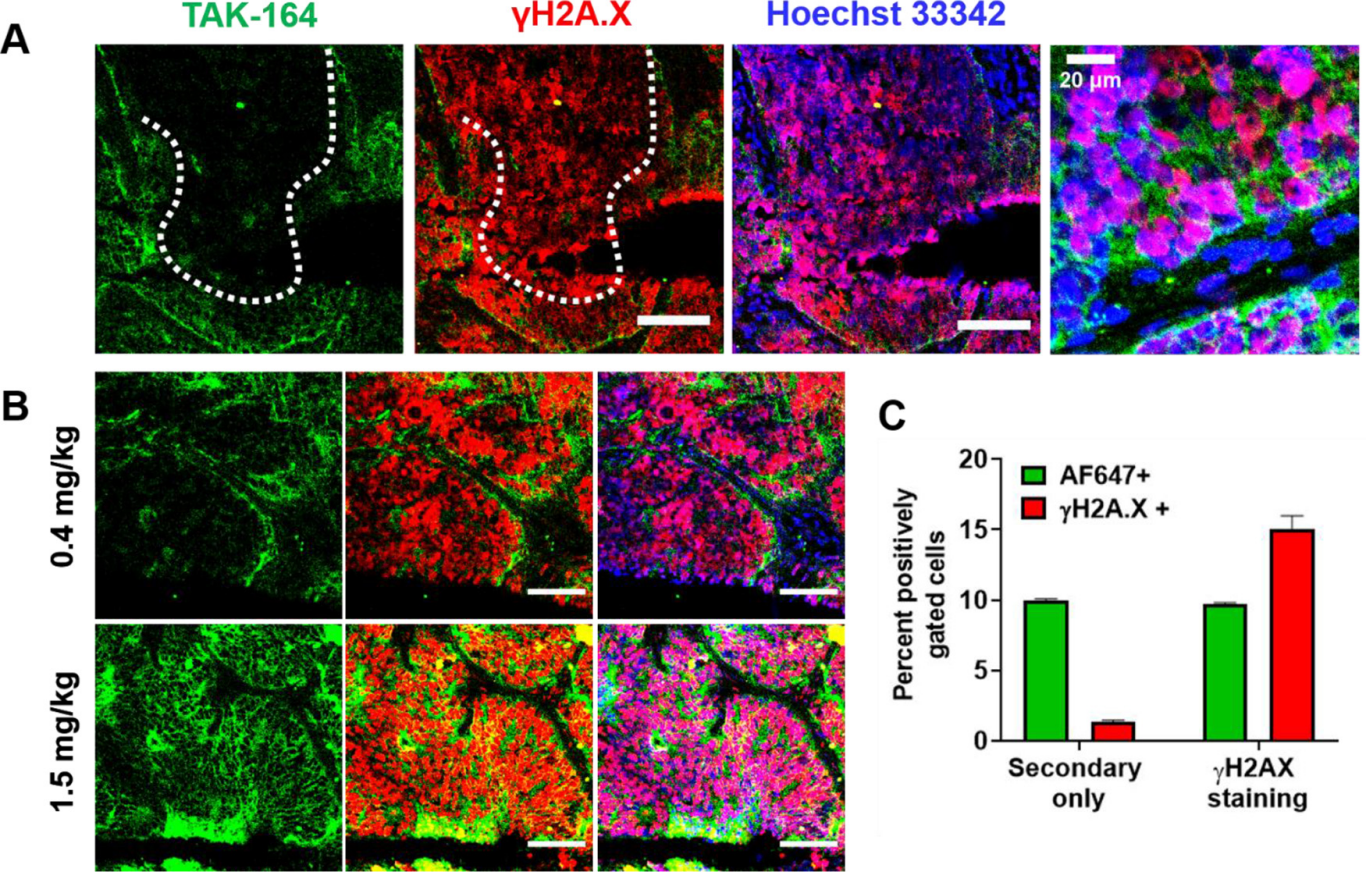
Single-cell resolution of DGN549 bystander penetration *in vivo*. (A) PHTX 11C tumors treated with 0.4 mg/kg of TAK-164 (*green*) showed heterogeneous, perivascular distribution of the ADC, targeting only a couple of cell layers, but γH2A.X signal (*red*) was observed much further than ADC fluorescence, and colocalized specifically with nuclear signal (*blue*). (B) ADC fluorescence and γH2A.X signal is elevated and more uniform throughout PHTX 11C tumors upon dose escalation (1.5 mg/kg vs 0.4 mg/kg). (C) Flow cytometry analysis of 0.4 mg/kg AF647-TAK-164 treated tumor digests show a greater fraction of cells showing γH2A.X signal compared to AF647 signal, indicative of bystander targeting by free DGN549. Scale bar = 100 μm.

Immunofluorescence images of PHTX 11C (Figure 3A, Supplementary Figure 8) show heterogeneous, perivascular TAK-164 distribution at 72 h, indicated by the short penetration front of AF647 fluorescent signal (*dotted white line*), ∼40 µm (Supplementary Figure 8). Comparatively, γH2A.X signal is detectable much farther than TAK-164 fluorescence. Similar analysis of tumors treated with fluorescent 5F9 (antibody only, no payload) showed little to no γH2A.X signal, indicating DGN549-mediated induction of H2A.X phosphorylation and bystander killing in TAK-164 treated tumors. High-magnification images revealed nuclear localization of DGN549-induced double-stranded DNA breaks (DSBs), indicated by overlap of γH2A.X and Hoechst 33342 signal. Dose-escalation in PHTX 11C tumor-bearing mice showed increased penetration of TAK-164, corresponding to increased γH2A.X signal uniformity and intensity (Figure 3B, Supplementary Figure 9). Flow cytometry analysis of single cells generated from treated PHTX 11C tumors confirmed a greater fraction of γH2A.X positive cells compared to AF647 positive cells (Figure 3C). Cells treated with secondary AF555-GAR only showed little γH2A.X signal. Together, these data provide cellular-level resolution evidence of spatial bystander killing [22] (SBE) with TAK-164. It is important to note that the reported percentage of γH2A.X positive cells via flow cytometry may not be directly comparable to the fraction of γH2A.X positive cells visualized via immunofluorescence histology for several reasons. γH2A.X signal and autofluorescence ranges in intensity, and the gating on flow cytometry was set conservatively to only capture cells above the highest levels of autofluorescence. Hypovascular and necrotic regions can also increase the percentage of γH2A.X negative cells via flow cytometry. Additionally, γH2A.X intensity varied within tumors, with some section showing less intense pharmacodynamic staining. These and other factors causing heterogeneity *in vivo* is a key reason we opted to perform distance quantification in the spheroid model.

### Fc-mediated bystander effects

**Fig. 4:**
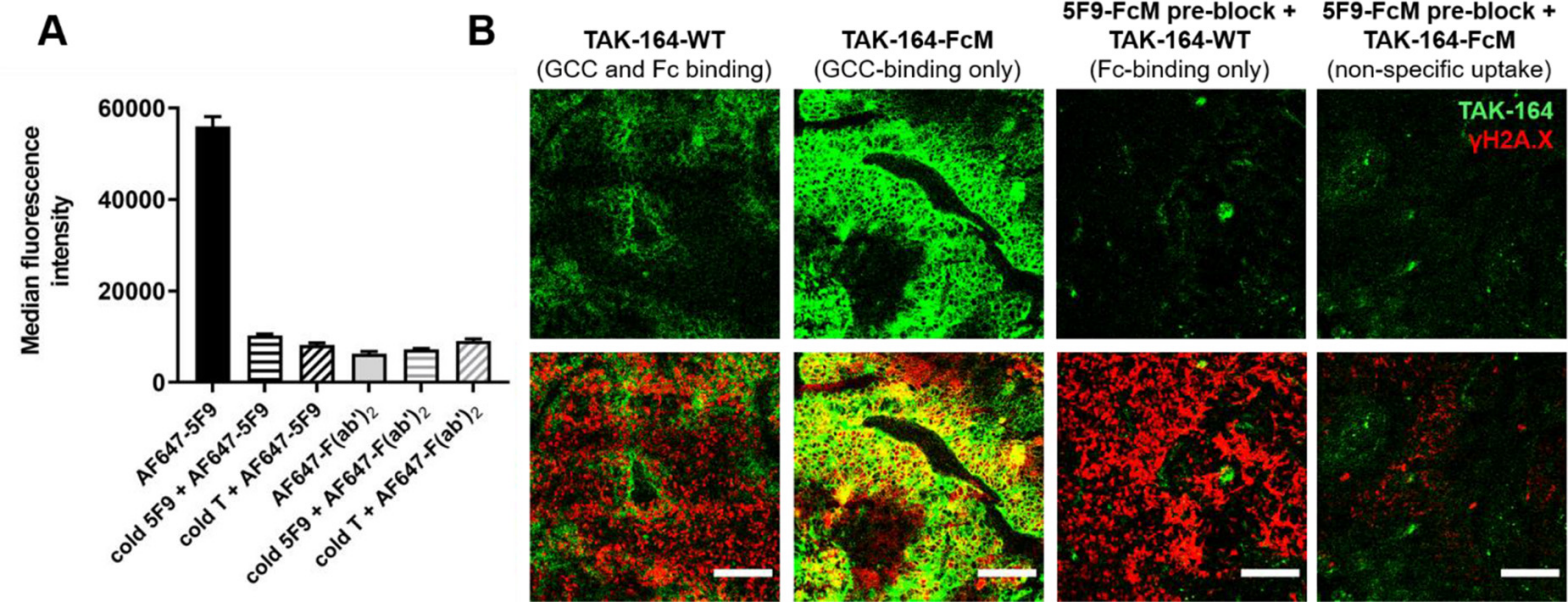
Fc-mediated uptake contributes to TAK-164 bystander effects. (A) Incubation of RAW macrophage cells with AF647-5F9 showed significant uptake which was diminished when preblocked with nonfluorescent (cold) 5F9 or with cold trastuzumab (T). Incubation with the 5F9 F(ab’)_2_ fragment which lacks Fc-domain also did not show uptake, with and without blocking, collectively indicating uptake in macrophages is specific and only FcγR-mediated. (B) PHTX 11C tumors with Fc-only uptake of TAK-164 showed γH2A.X staining comparable to GCC- and Fc-mediated uptake and GCC-only uptake, but little to no γH2A.X signal with nonspecific uptake only, indicating that Fc-mediated uptake by immune cells (e.g., TAMs) can efficiently process payload release and may contribute to ADC clinical efficacy. Note: Degree of labeling of TAK-164-FcM is higher (∼1.1) than TAK-164-WT (∼0.75). Scale bar = 100 μm.

The importance of tumor-associated macrophages (TAMs) in ADC efficacy is an ongoing debate, with contradictory evidence regarding the benefits of ADC uptake by TAMs. Immunofluorescence imaging of TAK-164 distribution in PHTX 11C (Supplementary Figure 10) and PHTX 17C (data not shown) tumors revealed uptake of intravenously administered fluorescent TAK-164/5F9 and binding of *ex vivo* 5F9 in macrophages. Given the previously reported tumor bystander killing from Fc-mediated uptake of ADCs by tumor-associated macrophages (TAM) [23], we sought to characterize the contribution of TAMs to bystander killing mediated by TAK-164 in PHTX 11C tumors. First, we determined the uptake in macrophages was Fc-mediated rather than expression of GCC on macrophages. RAW cells incubated with fluorescent 5F9 showed significant uptake of the antibody, which was blocked by nonfluorescent 5F9, nonfluorescent trastuzumab, or abrogated by removing the Fc domain of 5F9 (i.e., F(ab’)_2_ fragment). Blocking cells with nonfluorescent 5F9 did not further decrease signal of fluorescent F(ab’)_2_ 5F9 fragments, indicating no GCC binding was detectable (Figure 4A).

To evaluate the contribution of Fc-mediated uptake to TAK-164 bystander effects *in vivo*, we set-up 4 treatment groups in PHTX 11C tumor-bearing mice to capture – (1) GCC- and Fc- binding, (2) GCC only binding, (3) Fc-binding only, and (4) nonspecific uptake (Figure 4B). As expected, group 1 (GCC and Fc binding) showed heterogeneous ADC distribution (*green*) but more widespread γH2A.X staining (*red*). Group 2 with TAK-164-FcM (GCC only binding) showed better ADC intratumoral distribution compared to TAK-164-WT, indicating a redirection of ADC from Fc-mediated uptake to tumor cells. However, qualitatively γH2A.X bystander signal was not necessarily better (stronger or more widespread), indicating Fc-mediated uptake does not negatively impact ADC processing and payload release. Group 3 (Fc-only binding) mice were pretreated with 6mg/kg of cold 5F9-FcM, which blocked GCC receptors (effectively mimicking an antigen-negative system) but not FcγR on immune cells (e.g., TAMs). These tumors showed little TAK-164 fluorescence on tumor cells but widespread γH2A.X signal, indicating heterogeneous bystander effect/killing (HBE [22]) from ADC processing by immune cells expressing FcγR. This was confirmed by a lack of TAK-164 fluorescence or γH2A.X signal in Group 4 (nonspecific uptake) treated with cold 5F9-FcM and fluorescent TAK-164-FcM, resulting in no GCC or FcγR binding, and hence no cellular ADC processing to release free payload. These data provide directly observable confirmation that bystander effects help mitigate the impact of limited ADC delivery to heterogenous cell populations.

### High-throughput quantification of bystander payload penetration

PHTX tumor models provide a more clinically relevant platform for evaluation of pharmacokinetics and pharmacodynamics of *in vivo* ADC bystander killing compared to some cell-line derived xenografts, but they also contain multiple confounding factors for quantifying tissue penetration, including tumor vascular heterogeneity and systemic pharmacokinetics. *In vitro* 3-D tumor spheroids represent a high-throughput system used frequently to evaluate the distribution [[24], [25], [26], [27], [28]] and cytotoxicity [18,[29], [30], [31]] of therapeutic agents. Because HEK293-GCC xenografts exhibited heterogeneous ADC distribution *in vivo* (Figure 2), and HEK293 cells form spheroids [19,32,33], we used this as a model system to quantify tissue penetration of bystander payloads. Conditions for TAK-164 incubation were optimized to obtain peripheral ADC distribution using different time and concentration profiles (Supplementary Figure 1C). Spheroids treated for longer incubation durations (> 24 h) showed increasingly homogeneous distribution, while shorter durations (< 24 h) resulted in heterogeneous ADC fluorescence on the outer edge of the spheroid. Despite observing maximum γH2A.X signal at 72 h via flow cytometry (Supplementary Figure 5B), spheroids completely disintegrated by 60 h. Therefore, the ‘pulse-chase’ protocol included incubating spheroids for 9 h or 16 h to get heterogeneous ADC distribution but different total uptake (tuning bystander payload penetration), followed by a chase up to the end point of 54 h. Continuous incubation for 54 h served as a positive control and spheroids treated with 5F9 (no payload) as negative control for γH2A.X staining.

**Fig. 5:**
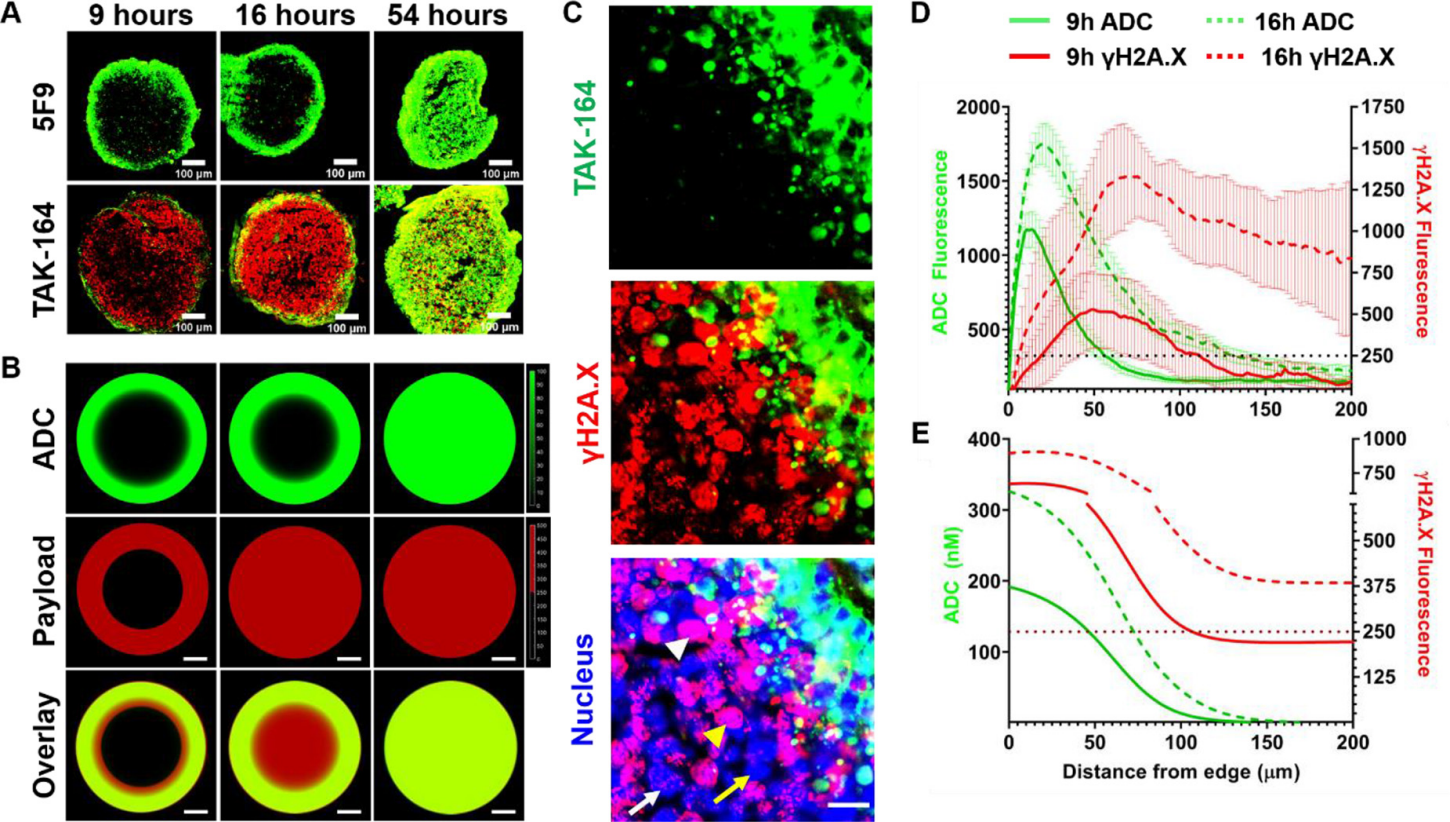
Quantification of DGN549 bystander penetration distance in HEK293-GCC spheroids. (A) Immunohistochemistry of spheroid sections treated with TAK-164 at varying conditions showed an increasing penetration front of DGN549-mediated DSBs, indicated by the spatiotemporal increase of γH2A.X signal in regions not targeted directly by the ADC. (B) Computational simulations showed a similar distribution pattern as the experiments spheroids. (C) High-resolution images confirm nuclear localization of the γH2A.X signal detected beyond ADC-targeted cells and revealed formation of various staining patterns observed in literature [34] (*white arrowhead = pan nuclear staining, yellow arrowhead = large foci, white arrow = small foci, yellow arrow = no foci*). Scale bar = 20 μm. (D) Euclidean distance mapping of tumor spheroids showed that γH2A.X signal (±SD) is observed well beyond the ADC-targeted (±SEM) cell layers, providing a quantitative estimate of DGN549 bystander penetration. The γH2A.X signal appeared to be reduced in ADC-targeted cells, though it is unclear if this is a result of peripheral free payload washout, signaling-mediated suppression of γH2A.X by 5F9 antibody, or some other factors (E) Radial plots of the computational results show similar ADC and DGN549 distribution patterns as the Euclidean map generated from experimental data.

Spheroids treated continuously for 54 h showed homogeneous ADC distribution (green) and γH2A.X staining (red), masking the distinction between direct cell killing and bystander killing (Figure 5A). Spheroids pulsed for 9 h had limited ADC uptake, so few cells in the center of the spheroid showed γH2A.X staining. However, several peripheral cell layers beyond those directly targeted by the ADC showed strong γH2A.X staining, indicative of bystander killing. The penetration front of bystander cell death increased all the way to the center of the spheroid when pulsed for 16 h with the ADC. High-resolution images of the spheroid edge after a 16-h pulse/chase condition confirmed the increased penetration of bystander killing relative to ADC fluorescence (Figure 5A, Supplementary Figure 11). There was a clear overlay of bystander γH2A.X signal with nuclear Hoechst 33342 (*blue*) and distinct patterns of γH2A.X signal, including pan-nuclear staining (*white arrowhead*), large widespread foci (yellow arrowhead), small distinct foci (white arrow), consistent with payload-induced dsDNA breaks detected by α-γH2A.X (Figure 5C). Euclidean distance map analysis [24] of experimental TAK-164 fluorescence and γH2A.X signal (Figure 5D) provided semi-quantitative confirmation of differential penetration distance of lethal payload concentration between 9 h pulse group (*N* =7, half-max signal R_Ab_ ∼50 µm, minimum R_γH2A.X_ ∼110 µm) vs 16 h pulse group (*N* =7, half-max R_Ab_ ∼75 µm, minimum R_γH2A.X_ ∼ 200 µm). Simulated spheroids (Figure 5B) pulsed for 9 h showed DGN549 bystander penetration a few cells beyond the ADC-targeted layer, while cells at the center remain viable. Simulations of spheroids pulsed for 16 h showed complete penetration of lethal payload concentration despite heterogeneous ADC distribution, while 54 h simulated spheroids were homogeneous in payload distribution as expected. Comparing the simulations to the Euclidean distance map, there was some discrepancy in the absolute concentrations estimated from an *in vitro* calibrations curve, likely a result of incomplete information regarding ADC and payload kinetic parameters. However, the plots of simulated ADC and payload concentration penetration (Figure 5E) revealed similar distribution trends as that in the Euclidean distance map of experimental spheroids (Figure 5D),

## Discussion

ADC payload discovery and design has witnessed rapid growth in recent years, with reports of several new bystander payloads. However, the quantification and relative impact of bystander effects remain difficult to study. The concept of bystander cell killing has roots in radioimmunotherapy (RIT), where radiolabeled antibodies could kill untargeted cancer cells adjacent to the targeted cells due to bystander radiation exposure [35]. Similar bystander cell killing was observed with the development of second- and third-generation ADCs carrying maytansinoids (e.g., s-methyl DM4), auristatin (e.g., monomethyl auristatin E), and DNA crosslinking/alkylating payloads (e.g., pyrrolobenzodiazepenis), wherein the released payload could diffuse from an ADC-targeted cell to adjacent targeted/untargeted cells, often enhancing cell death. Despite the shared name, these ADC bystander effects are more similar to direct ‘crossfire’ killing of RIT than radiobiology ‘bystander effects’ (e.g., mediated by reactive oxygen species/ROS) since noncleavable payloads that cannot diffuse to other cells generally do not demonstrate bystander cell killing [[36], [37], [38], [39], [40]]. While literature and data in this work shows that bystander killing is mediated directly by the released payload, it is important to note the potential for immunogenic cell death (ICD) where molecules (e.g., Damage Associated Molecular Patterns/DAMPs) release from dead and dying cells could trigger immune activation [41,42], and contribute to clinical efficacy. Currently, 5 of the 9 FDA-approved ADCs, and several others in clinical trials, employ payloads with reported bystander effects, but the tissue penetration distance of this effect remains unclear.

Bystander effects can enhance the efficacy of ADCs by compensating for intratumoral ADC distribution heterogeneity (spatial bystander effects, SBE) and/or receptor expression heterogeneity (heterogeneous bystander effects, HBE) [22]. To date, both effects have largely been explored in bulk systems evaluating the survival of admixed tumors *in vivo* or co-culture cells *in vitro*. For example, Kovtun et al. demonstrated that in mixed-model tumors with antigen-positive and antigen-negative cells, treatment with disulfide-linked huC242-DM1 (releasing cell-permeable metabolites [43]) led to necrosis in both cell populations (i.e., HBE). This was in stark contrast to treatment with huC242-SMCC-DM1 (and nonpermeable metabolite), which caused necrosis in only antigen positive cells [37]. Similarly, Hingorani et al. showed more diffuse pS10 Histone H3 (pharmacodynamic marker of mitotic arrest) that was not restricted to HER2-positive tumor regions when treated with Trastuzumab-MMAE (bystander payload) compared to Trastuzumab-MMAF (nonbystander payload) [36]. More recently, Ilovich et al. demonstrated bystander penetration of MMAE using dual-isotope cryoimaging in xenograft tumors treated with TAK-264 [10]. The study showed that in GCC-positive tumors, only 0.8% of voxels had the payload and antibody radiolabels colocalized compared to 15% voxel colocalization in GCC-negative tumors, indicating considerable ^3^H-MMAE diffusion through the tumor beyond ADC-targeted cells (i.e., SBE). This study provided one of the highest spatial-resolution (tissue-level) imaging studies of SBE and direct visualization of bystander payload penetration. However, detailed studies on bystander payload tissue penetration, indicative of their ability to compensate for heterogeneous ADC distribution, remain sparse.

Bystander killing efficiency is dependent on the interplay between cell uptake of released payload and the extracellular payload diffusion, with balanced rates allowing even distribution without significant tumor washout. Cell permeability and mass transport kinetics are influenced by distinct physicochemical properties, and even subtle differences in these properties can alter the cytotoxic/bystander potential of a molecule [44,45]. For example, maytansinoid metabolites Lys-SMCC-DM1 and S-methyl DM4 exhibit a marked difference in cellular behavior, with the more lipophilic S-methyl DM4 exhibiting pronounced cellular permeability and bystander cell killing [43] compared to the more charged and bulky Lys-SMCC-DM1. However, oxidation of S-methyl DM4 to S-methyl DM4 sulfoxide can revert the potency and bystander potential nearly 30-fold [46].

Moderately lipophilic payloads possess desirable tissue penetration properties based on computational simulations, matching the cell uptake rate with extracellular diffusion and resulting in the maximum number of cells receiving a lethal dose prior to washout of the payload (Figure 1A, B). However, these predictions are difficult to validate experimentally. The strong dependence of bystander behavior on payload properties precludes the use of fluorescent-tagging of payloads for direct tracking, which alters the payload pharmacokinetics. Radiolabeling does not provide the sensitivity, resolution, and in some cases, tolerability, needed at clinically relevant doses of DNA-interacting payloads. Given the ultra-high potency of these payloads, even an extremely low drug concentration (below the limit of detection for radioimaging) is sufficient to induce a pharmacodynamic response. For DNA-interacting payloads that cause DSBs, a key signaling response is the γ-phosphorylation of histone H2A.X [47], which can be detected sensitively with immunofluorescence imaging (Supplementary Figure 4). Therefore, we used indirect imaging of a pharmacodynamic marker to track bystander tissue penetration with cellular resolution.

To visualize these effects in 3D tissue culture and *in vivo*, we chose 2 model systems that resulted in heterogeneous ADC distribution (Figure 2), physically separating direct cell killing (i.e., cells directly targeted by ADC) from bystander cell killing based on proximity to blood vessels (*in vivo*) or cell culture media (*in vitro*). Transfected HEK-293 cells were used for tumor spheroid studies *in vitro*, and a primary human tumor xenograft model (PHTX 11C) was selected for *in vivo* studies. Using the γH2A.X DNA-damage marker, bystander effects were readily apparent in the PHTX model (Figure 3A). The heterogeneous distribution of the fluorescently labeled ADC contrasted strongly with the more uniform γH2A.X staining throughout the regions of cancer cells. Increasing doses *in vivo* also correlated with stronger γH2A.X staining (Figure 3B), similar to *in vitro* staining (Supplementary Figure 4B), indicating the marker could serve as a semi-quantitative measure of payload delivery. Single cell digests of tumors showed a considerable fraction of γH2A.X positive cells lacked ADC signal (Figure 3C), consistent with the histology imaging indicating bystander killing.

Immunofluorescence imaging of TAK-164 pharmacokinetics showed measurable uptake in stromal cells, most notably tumor associated macrophages (TAMs), an abundant immune cell population in tumors [[48], [49], [50]]. TAMs play an important role in tumor immune effects [42,51,52]. Additionally, Li et al. reported that nonspecific hIgG-vc-MMAE resulted in similar efficacy as tumor-specific αCD30-vc-MMAE in preclinical tumors, owing to the Fc-mediated TAM uptake of the tumor nonspecific ADC and subsequent bystander efficacy. Indeed, in their study, a direct correlation was observed between the degree of TAM infiltration (which can be variable) and efficacy of the tumor nonspecific ADC [23], indicating the potential for TAMs to contribute to ADC efficacy. More recently, Staudacher et al. show that phagocytosis of dead tumor cells targeted with a PBD-based ADC by TAM resulted in bystander killing of both TAMs and bystander tumor cells, enhancing efficacy [53]. However, evidence with Brentuximab vedotin (MMAE) also suggests that Fc-mediated uptake of the ADC results in incapacitation of TAMs, limiting immune function in tumor suppression [54]. Thus, whether Fc-mutations limiting immune-cell uptake of the ADC are beneficial remains an important but underappreciated factor in clinical ADC development. While TAM binding to intact Fc antibody domains likely occurs in most tumors, it was more apparent here due to the more modest expression levels of GCC relative to highly expressed targets such as HER2.

To test if the Fc-mediated TAM uptake played a role in payload release, we first examined the mechanism of ADC uptake (Figure 4A). Uptake in RAW cells could be blocked by a target-specific antibody (5F9), a nonspecific antibody (trastuzumab), or by removing the Fc domain (F(ab’)_2_ fragment). No difference was seen by further blocking F(ab’)_2_ uptake with 5F9 antibody. Therefore, the mechanism appeared to be exclusively Fc-mediated internalization (versus GCC-mediated uptake). *In vivo*, a combination of mutant Fc antibodies and ADCs (that do not bind Fc-gamma receptors) was used to direct ADC to cancer cells, immune cells, or both (Figure 4B). Fc-mutant TAK-164 resulted in better intratumoral penetration, presumably because all ADC that would have been taken up by FcγR-expressing immune cells (e.g., TAMs) was redirected to GCC, improving penetration. While both Fc-mutant and wild-type Fc TAK-164 showed strong γH2A.X staining at low doses and similar efficacy at high doses (data not shown), TAK-164 uptake in immune cells mediated solely via Fc-domain (no GCC- tumor uptake) also resulted in significant γH2A.X staining. This data suggests bystander killing from Fc-mediated uptake in immune cells could partially contribute to efficacy even when artificially directing all the ADC to immune cell such as TAMs. Additionally, these data demonstrate that Fc-mediated heterogeneous bystander effects can also compensate for heterogeneous or low antigen expression (mimicked here by preblocking all GCC receptors with nonfluorescent 5F9).

Efficient bystander killing in PHTX tumors by DGN549 (DNA-interacting payload) is consistent with previous model predictions, but many confounding factors (e.g., multiple cell types, plasma clearance, heterogeneous delivery) make it difficult to quantify tissue penetration *in vivo*. Therefore, we used *in vitro* HEK293-GCC tumor spheroids to quantify DGN549 tissue penetration relative to ADC fluorescence (Figure 5). Pulses of 9 and 16 h resulted in peripheral ADC uptake in spheroids, followed by chasing the spheroids with media until 54 h to allow deeper payload diffusion. Pharmacodynamic staining showed a steep gradient towards the center of spheroids with the 9-h pulse, indicating the payload concentrations were not sufficiently high to cause DSBs deep in the spheroids. In contrast, the 16-h pulse group showed γH2A.X staining almost to the spheroid center, consistent with efficient tissue penetration of lethal payload concentrations. Both of these observations are consistent with limited washout from the tissue due to a balance between cellular uptake and diffusion as predicted by the computational model.

While pharmacodynamic staining is a valuable tool in evaluating how far DNA-interacting bystander payloads can penetrate in a tumor, it is critical to note that this technique has several limitations. The detection of γH2A.X is not an absolute indicator of cell death [55] and cannot be directly equated to efficacy. γH2A.X indicates the recruitment of DNA repair enzymes to the sites of DSBs, evidenced by increased radiosensitivity in H2A.X deficient mice [56], and onset of apoptosis depends on the degree of damage. Likewise, the relationship between payload concentration and pharmacodynamic signal can be nonlinear. However, the pattern of γH2A.X can be informative, with the apoptotic γH2A.X ring [57] and pan-nuclear staining [34] indicating excess DSBs, triggering apoptosis over cell repair. Additionally, not all γH2A.X signal arises from exogenously induced DSBs. Mitotic phosphorylation of H2A.X has been observed even in the absence of treatment [58] (Supplementary Figure 4, Supplementary Figure 5A, yellow arrowhead) possibly due to formation of endogenous DSBs during replication and transcription [59]. Although antibodies themselves are not expected to generate DSBs, trastuzumab has been observed to both enhance [60] and suppress [61] γH2A.X signal, likely due to overlap in signaling pathways. Appropriate controls must be employed to verify observed γH2A.X is payload-induced (here we used 5F9 antibody as a negative control). Finally, γH2A.X accumulation is dose, time, cell-cycle, and mechanism of DNA-damage dependent [55,62]), and is also transient, dissipating after peak accumulation [12,63,64] (Supplementary Figure 12). Therefore, care should be taken in designing experiments that utilize γH2A.X has a pharmacodynamic marker for ADC payload penetration.

This work builds on established tools using as pharmacodynamic markers and 3D cell culture to quantitatively measure the physical penetration depths to which bystander ADC payloads can still exert their cytotoxic effect, providing a rapid, high-throughput, and physiologically relevant platform to enhance the field's fundamental understanding of ADC bystander effects. Future directions of this work include evaluating panels of ADC payloads to develop a quantification metric, similar to the Damköhler number [9], that correlates intrinsic physicochemical and molecular payload properties to anticipated *in vivo* bystander penetration to rapidly quantify bystander killing potential. Additionally, the use of multicellular tumor spheroids to mimic a complex tumor environment, consisting of antigen negative cells [37], immune cells [65] such as tumor-associated macrophages and dendritic cells, stromal-cells and ECM (e.g., DAaRTs [66]), etc. can help dissect role in bystander delivery of ADC payloads in a nonhomogeneous tumor cell environment and also the activation of the immune cells themselves by the payload (1) to enhance efficacy.

## Conclusions

Overall, we show that pharmacodynamic staining can be a valuable tool to visualize ADC bystander payload tissue penetration *in vitro* and *in vivo*, particularly for agents that are difficult to image with radiolabeling approaches. The DGN549 payload from TAK-164 penetrated tissue ∼100 to 200 microns beyond the antibody-targeted cells as evidenced by a pharmacodynamic response. This distance is similar to computational model predictions, where the payload penetrates deep into the tumor while maintaining sufficient cell uptake because cellular uptake is balanced with diffusion to avoid significant washout from the tissue. Furthermore, pharmacodynamic staining was used to evaluate additional contributions to ADC efficacy, such as payload release and bystander effects via Fc-mediated ADC uptake in immune cells (most likely TAMs). Together, this multiplexed *in silico-in vitro-in vivo* platform can help quantify payload bystander potential with cellular resolution.

## Credit author statement

*Eshita Khera*: Investigation, methodology, visualization, software, writing – original draft. *Cornelius Cilliers*: Investigation, writing – review and editing. *Michael D. Smith*: Investigation, writing – review and editing. *Michelle L. Ganno*: Investigation, writing – review and editing. *Katharine C. Lai*: Investigation, writing – review and editing. *Thomas A. Keating*: Investigation, writing – review and editing. *Anna Kopp*: Investigation. *Ian Nessler*: Software. *Adnan O. Abu-Yousif*: Conceptualization, writing – review and editing, *Greg M. Thurber*: Conceptualization, supervision, writing – original draft.

## Funding

This work was supported by Takeda, NIH R35 GM128819 (GMT), and the National Cancer Institute of the National Institutes of Health under Award Number P30CA046592 by the use of the following Cancer Center Shared Resource(s): histology.

## Conflict of interest

MDS, MLG, and AOA were employed by Takeda, and KCL, and TAK were employed by Immunogen during the study. GMT sits on the Scientific Advisory Board of Advanced Proteome Therapeutics.

## Appendix Supplementary materials

Image, application 1
