# Supplementary material for: Quantifying ADC bystander payload penetration with cellular resolution using pharmacodynamic mapping
Source: Neoplasia. 2020 Dec 29;23(2):210–21. doi: 10.1016/j.neo.2020.12.001 (PMC7779838; doi:10.1016/j.neo.2020.12.001)
Supplement: Supplementary file 1 [file mmc1.docx]

**Quantifying ADC bystander payload penetration with cellular resolution using pharmacodynamic mapping**

Eshita Khera^a^, Cornelius Cilliers^a^, Michael D. Smith^c^, Michelle L. Ganno^c^, Katharine C. Lai^d^, Thomas A. Keating^d^, Anna Kopp^a^, Ian Nessler^a^, Adnan O. Abu-Yousif^c^, Greg M. Thurber^a,b^

^a^ Department of Chemical Engineering, University of Michigan, Ann Arbor, MI 48109

^b^ Department of Biomedical Engineering, University of Michigan, Ann Arbor, MI 48109

^c^ Millennium Pharmaceuticals Inc.*, Cambridge, MA, 02319

* *a wholly owned subsidiary of Takeda Pharmaceutical Company Limited*

^d^ Immunogen Inc., Waltham, MA, 02451

**Supplementary Data**

Bystander payload penetration computational model framework – Figures S1-S3

- Model description
- Estimating TAK-164 cellular internalization rate
- Estimation of DGN549-DNA reaction kinetics
- γH2A.X calibration curve
- Estimating DGN549 distribution kinetics
- Model Equations
- Boundary Conditions
- Model Parameters – Table S1

Supplemental Figures – S4 to S11

References

**Bystander payload penetration computational model framework**

Spatiotemporal ADC and payload distribution in the spheroids were simulated using a computational spheroid model consisting of partial differential equations(1). Briefly, a 1-D cylindrical Krogh cylinder model (radial only) capturing ADC transport kinetics (systemic pharmacokinetics, extravasation, intratumoral diffusion, receptor binding, cellular internalization, and lysosomal degradation) and payload transport kinetics (lysosomal release, DNA binding, cellular escape/internalization, and intratumoral diffusion) was modified to describe distribution in *in vitro* tumor spheroids (Supplementary Figure 1A).

**
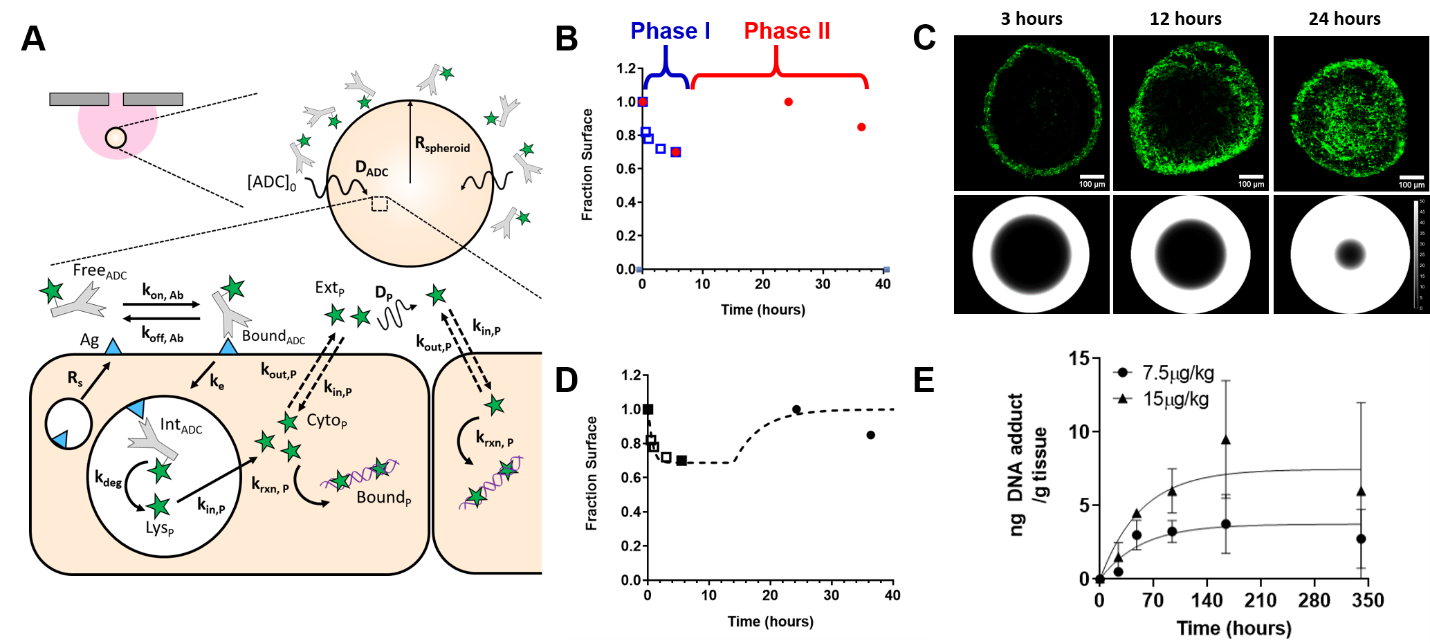
Supplementary Figure 1. Development of the bystander payload spheroid model framework. (A)** Schematic of mechanistic tumor spheroid simulation model adapted from the Krogh cylinder model**. (B)** In vitro data with HEK293-GCC cells shows non-linear internalization kinetics of TAK-164, marked by an acute, rapid internalization Phase I that results in moderate receptor downregulation, followed by a slow internalizing Phase II that allows recovery of surface receptor. **(C)** 3D temporal distribution data for 5F9 in tumor spheroids **(D)** A two-phase step-function (*dotted line*) with internalization rates optimized using 2D internalization data and 3D spheroid distribution data was derived and used for subsequent simulations. **(E)** Two-ligand concentration association rate fit for DGN549-DNA adduct formation, performed using previously published data(2).

*Estimating TAK-164 cellular internalization rate*

*In vitro* experiments to measure TAK-164 internalization revealed the influence of a complex biological binding response resulting in non-linear kinetics. TAK-164 undergoes acute rapid receptor internalization on HEK293-GCC cells, activated by binding of TAK-164 to GCC. This results in partial downregulation of receptor expression (~30-50%) at early time points, but slowly recovered by 24 hours post incubation. Consequently, a constant first order internalization rate could not be estimated for TAK-164. Instead, a two-phase internalization rate was derived involving a step-function with an acute rapid internalization and downregulation phase, followed by a recovery phase with slower internalization. Internalization rate constants for Phase I was determined using the TAK-164 internalization data in monolayer cells (Supplementary Figure 1B), while that for Phase II was fit using both TAK-164 internalization data and spatiotemporal distribution profiles in tumor spheroids (Supplementary Figure 1C), to generate the optimized non-linear kinetics capturing TAK-164 internalization (Supplementary Figure 1D), which was used in subsequent simulations. It is important to note here that while this internalization step-function may capture the non-linear kinetics for 2D cells, it does not perfectly translate in a deterministic Krogh cylinder model. The Krogh cylinder model is not object-oriented, and the transition from a rapidly internalizing receptor to a slow-internalizing receptor is applied globally in the spheroid model i.e. all cells (regardless of whether they have encountered an ADC or not) transition from Phase I to Phase II simultaneously, as opposed to the more biologically likely effect that only cells that encounter the ADC are triggered to have non-linear internalization kinetics.

*Estimating DGN549-DNA reaction kinetics*

Since little direct data is available on the kinetics of the steps between lysosomal payload escape and DGN549-DNA adduct formation, we simplified the computational model to approximate effective reaction rate (k_rxn_) between free intracellular payload and DNA (i.e. group together nuclear membrane diffusion and DGN549-DNA binding) using available literature data on DNA-payload adduct formation. Li et al. utilized an LC/MS method to quantify DNA adduct formation with various IGNs (class of DNA-interacting payloads that includes DGN549) in tumors and performed a dose- and time-dependent DNA adduct formation data (data points replotted in Supplementary Figure 1E). Using this data along with an approximation of the free payload concentration in the cell, we performed the two-ligand concentration association kinetics analysis in GraphPad Prism 8 to extract an estimated reaction rate between free intracellular DGN549 and DNA (fitted lines in Supplementary Figure 1E, k_rxn_ ~ 0.032/M⋅s). This slow reaction rate is consistent with observations by Singh et al. that the formation of adducts between the monoamine IGN1 (similar to DGN549) and DNA occurs over the course of 1-2 days. Additionally, they observed little to no release of the free payload from genomic material without nucleases, indicating that adduct formation is slow and irreversible(3), which was incorporated into our simulations.

*In vitro γH2A.X calibration*

We have previously reported our Krogh cylinder bystander model simulation results in the form of the total intracellular payload concentration, utilizing a ‘therapeutic threshold’ cut-off concentration for microtubule inhibitors to assess the bystander penetration front of the payload(1). In order to compare the simulations to the experimental spheroid data, we performed a calibration between the simulated intracellular payload concentration and the observed γH2A.X signal to generate a sigmoidal function that correlates intracellular payload concentration to γH2A.X signal. Briefly, we analyzed dose-dependent cell γH2A.X immunohistochemistry data (Supplementary Figure 4) by first generating a binary mask on the Hoechst 33342 positive cells. The outline of cells from the binary mask were overlaid on the γH2A.X channel to calculate the average γH2A.X signal for 300-500 cells in each concentration group (Supplementary Figure 2A). Using a cellular ordinary differential equations (ODE) model which used the same kinetic parameters as the spheroid model, we converted the extracellular TAK-164 concentration (Supplementary Figure 2B) to intracellular payload concentration (Supplementary Figure 2C) and plotted both against the average γH2A.X signal for each treatment group. The γH2A.X calibration curves were plotted against viability data performed for the same duration (~2.5 days) to extract the ‘therapeutic threshold’ γH2A.X signal the correlates to 100% cell death. We found the minimum “therapeutic threshold” to correspond to C_min_ ~ 30nM and γH2A.X_min_ ~50 fluorescence units and use this as a cutoff to qualitatively compare experimental spheroid images to simulations results. For a quantitative comparison, we converted the simulation payload concentration to γH2A.X signal using the sigmoidal function (*red*) in Supplementary Figure 2C.


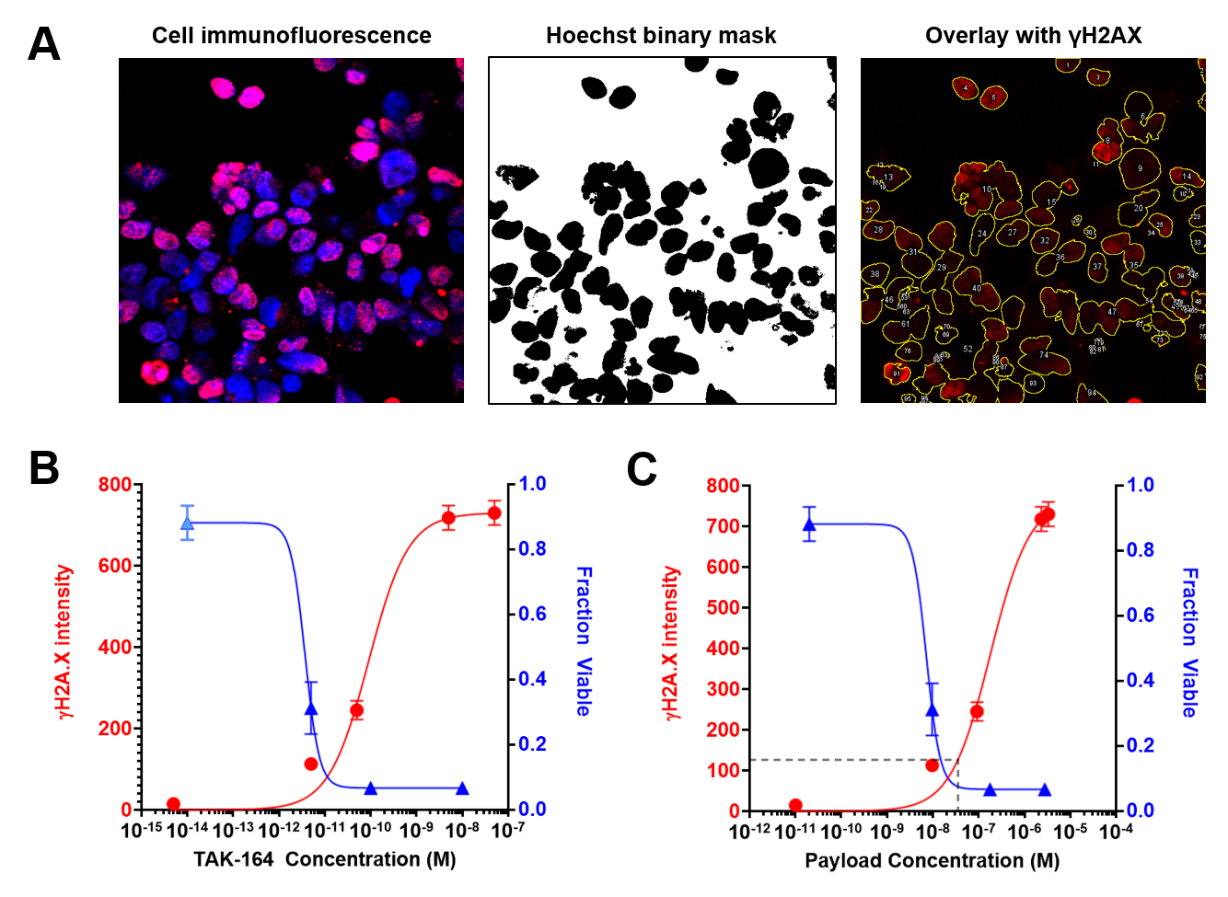


**Supplementary Figure 2. γH2A.X calibration analysis. (A)** Confocal images of monolayer cells treated with increasing concentrations of TAK-164 were analyzed using ImageJ to measure the average γH2A.X per treatment group. **(B)** Correlation plot between extracellular TAK-164 concentration, measured γH2A.X signal intensity and cellular viability. **(C)** Correlation plot between intracellular DGN549 concentration estimated from extracellular TAK-164 concentration using a cellular ODE model, measured γH2A.X signal intensity and cellular viability.

*Estimating DGN549 distribution kinetics*


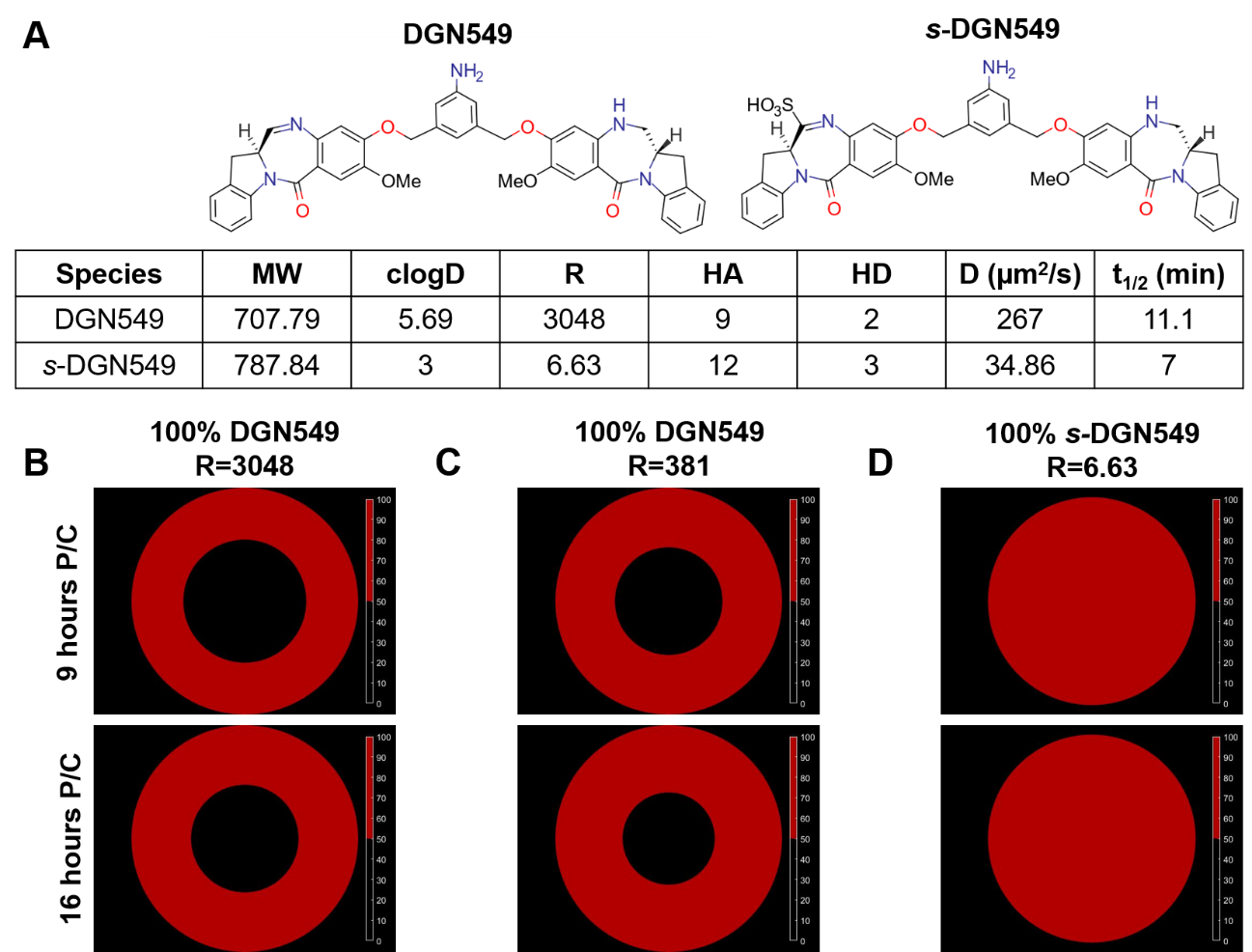


**Supplementary Figure 3. Estimating payload distribution kinetics. (A)** Estimated physicochemical properties of DGN549 and sulfo-DGN549 using previously published methodology(1). Predicted γH2A.X signal of (B) 100% payload species being DGN549 with estimated R =3048, **(C)** 100% payload species being DGN549 with R = 381 (measured for BODIPY650-olaparib(4)), **(D)** 100% payload species being *s*-DGN549 with estimated R = 6.63

Due to the lack of absolute/measured rates in the literature for the multi-step process of payload release, targeting of the nucleus, binding, and covalent modification, the overall rate of DGN549-DNA adduct formation was estimated based on empirical observations and literature rate data for individual steps. Like most other ADCs containing DGN549(5,6), conjugation of generation of TAK-164 is performed using a fully sulfonated form of DGN549 (hereon referred to as *s*-DGN549) due to high lipophilicity/low aqueous solubility of un-sulfonated DGN549(5) (hereon referred to as DGN549). Structural analysis of DGN549 and s-DGN549 using MarvinSketch (ChemAxon) confirm that while both forms have similar molecular weight, DGN549 is extremely lipophilic, with a calculated logD of 5.69 (Supplementary Figure 3A). Such a high clogD results in an extremely large calculated partition coefficient (R), which when used to scale the payload cellular escape rate (k_out,P_) and the extracellular diffusion rate (D_eff,P_)(1) predicts negligible escape of the DGN549 payload in the experimental timeframe (Supplementary Figure 2B). This contradicts both spheroid and PHTX tumor data that demonstrates considerable bystander killing. While scaling k_out,P_ and D_eff,P_ with the calculated partition coefficient with this model framework has reasonably captured the expected behavior of a range of molecules(1,7), it has not previously been applied to extremely lipophilic molecules and may not accurately capture experimental partition coefficient for extremely lipophilic molecules. For example, olaparib-BODIPY650 is extremely lipophilic (clogD = 6) but was found to have an experimental partition coefficient of 381(4), nearly 10-fold lower than that predicted for DGN549. However, even the relatively lower olaparib-BODIPY650 partition coefficient did not sufficiently change the predicted behavior of DGN549 (Supplementary Figure 3C), even qualitatively, indicating additional experimental effects not being captured by the model. In particular, cellular catabolism studies of TAK-164 indicate the release of both DGN549 and s-DGN549 as free payloads, with both forms existing in equilibrium(8). The cellular influx rate (k_in,P_) for DGN549 and s-DGN549 are estimated from a local-fit analysis(1) to be similar, but their partition coefficients vary greatly, which influences the effective cellular escape rate and diffusion coefficient, and overall distribution patterns (Supplementary Figure 3D). However, no information is readily available on what fraction of the free payload released is DGN549 vs *s*-DGN549, which limits the specificity of parameters that can be input into the model. Instead, we opted to empirically modify the effective partition coefficient (R_eff_ =18, corresponding to clogD ~ 3) to match the observed experimental pattern of payload distribution, noting that this R_eff_ could arise from a combination of experimental effects. Using this R_eff_, simulations of ADC and payload distribution in spheroids mimicking the described pulse/chase set-up qualitatively match experimental observations (Figure 5B). Quantitative comparisons showed an overall agreement in trends, though the absolute γH2A.X calculation did not always perfectly match the experimental γH2A.X signal intensity. This could be a result of the simplified TAK-164 internalization kinetics used for this model, which affects the overall intracellular payload concentration and can influence the absolute γH2A.X calculation while maintaining a similar trend.

*Model Equations*

1. **Free ADC**

$$\frac{\partial C_{ADC}}{\partial t}= D_{eff}\left( \frac{1}{r^{2}}\frac{\partial}{\partial r}\left( r^{2}\frac{\partial C_{ADC}}{\partial r} \right) \right)-k_{on}\frac{C_{ADC}}{\varepsilon}T_{free}+k_{off}B_{ADC}$$

2. **Free Target**

$$\frac{\partial T_{free}}{\partial t}= R_{s}-k_{on}\frac{C_{mab}}{\varepsilon}T_{free}-k_{on}\frac{C_{ADC}}{\varepsilon}T_{free}+k_{off}B_{ADC}-k_{e}T_{free}$$

3. **Bound ADC**

$$\frac{\partial B_{ADC}}{\partial t}=k_{on}\frac{C_{ADC}}{\varepsilon}T_{free}-k_{off}B_{ADC}-k_{int}B_{ADC}$$

4. **Internalized ADC**

$$\frac{\partial C_{int,ADC}}{\partial t}=k_{int}B_{ADC}- k_{deg}C_{int,ADC}$$

5. **Extracellular payload**

$$\frac{\partial C_{ext,P}}{\partial t}=D_{eff,P}\left( \frac{1}{r}\frac{\partial}{\partial r}\left( r\frac{\partial C_{ext,P}}{\partial r} \right) \right) -k_{in,P}\frac{1-\varepsilon_{P}}{\varepsilon_{P}}C_{ext,P}+k_{out,P}C_{int,SP}$$

6. **Intracellular payload**

$$\frac{\partial C_{int,P}}{\partial t}= k_{in,P}\frac{1-\varepsilon_{P}}{\varepsilon_{P}}C_{ext,P}-k_{out,P}C_{int,P}-\frac{k_{on,P}}{\left( 1-\varepsilon_{P} \right)R}\left( P_{target}- C_{bound,P} \right)C_{int,P}+k_{off,P}C_{bound,P}+ k_{in,P}C_{lyso,P}$$

7. **Bound payload**

$$\frac{\partial C_{bound,P}}{\partial t}=\frac{k_{rxn,P}}{\left( 1-\varepsilon_{P} \right)R}\left( P_{target}- C_{bound,P} \right)C_{int,P}$$

8. **Lysosomal payload**

$$\frac{\partial C_{lyso,P}}{\partial t}= DAR*k_{deg}C_{int,ADC}{- k}_{in,P}C_{lyso,P}$$

*Boundary Conditions*

1. Neumann Boundary Condition at spheroid center

$D_{eff}\left. \frac{dC_{free}}{dr} \right|_{r=R_{spheroid center}}=0$

2. Dirichlet Boundary Condition at spheroid edge

$$\left. C_{ext, P} \right|_{r=R_{spheroid edge}}=0$$

$\left. C_{free, ADC} \right|_{r=R_{spheroid edge}}={[C]}_{0}$

**Supplementary Table 1 – Simulation model parameters.**

| **Parameter** | **Value** | **Unit** | **Description** | **Reference** |
| --- | --- | --- | --- | --- |
| R_spheroid_ | 275 | μm | Spheroid radius |  |
| D_ADC_ | 10 | μm^2^/s | Antibody/ADC diffusivity | (9) |
| ε | 0.15 | ND | Tumor void fraction | (10) |
| [Ag]_0_ | 83.3 | nM | Initial antigen concentration (100,000 GCC/cell) | Measured |
| R_s_ | k_e_*[Ag]_0_ | μM/s | Antigen recycle rate | Fit |
| k_on, Ab_ | 1x10^5^ | M^-1^s^-1^ | Antibody/ADC binding rate | Typical Estimate |
| k_off, Ab_ | 6x10^−6^ | s^-1^ | Antibody/ADC dissociation rate | k_on, Ab_*K_d_ |
| K_d_ | 60 | pM | Antibody/ADC dissociation constant | Measured |
| k_e,I_ | 3x10^−4^ | s^-1^ | Antibody/ADC internalization rate | Fit |
| k_e,I,bound_ | 4.6x10^−4^ | s^-1^ | Antibody/ADC internalization rate |
| k_e,II_ | 4.8x10^−5^ | s^-1^ | Antibody/ADC internalization rate |
| k_e,I,bound_ | 4.8x10^−5^ | s^-1^ | Antibody/ADC internalization rate |
| k_deg_ | 8.0x10^−6^ | s^-1^ | ADC lysosomal degradation rate | Measured |
| DAR | 3 | ND | Drug to antibody ratio |  |
| k_in,P_ (DGN549) | 1.05x10^−3^ | s^-1^ | Payload influx rate | Estimated(1) |
| k_out,P_ (DGN549) | k_in,P_/(1+ R_eff_) | s^-1^ | Payload efflux rate | Estimated(1) |
| D_P_ (DGN549) | 267/(1+R_eff_) | μm^2^/s | Payload diffusion coefficient | (1,11) |
| R_eff_ | 18 | m/s | Payload partition coefficient | Estimated |
| k_rxn, P_ | 0.032 | M^-1^s^-1^ | Payload binding rate | [Estimated](#_ENREF_77)(2,3) |
| ε_p_ | 0.44 | ND | Cell void fraction | (1) |
| P_target_ | 71 | μM | DNA concentration | (7) |

**Supplementary Figures**


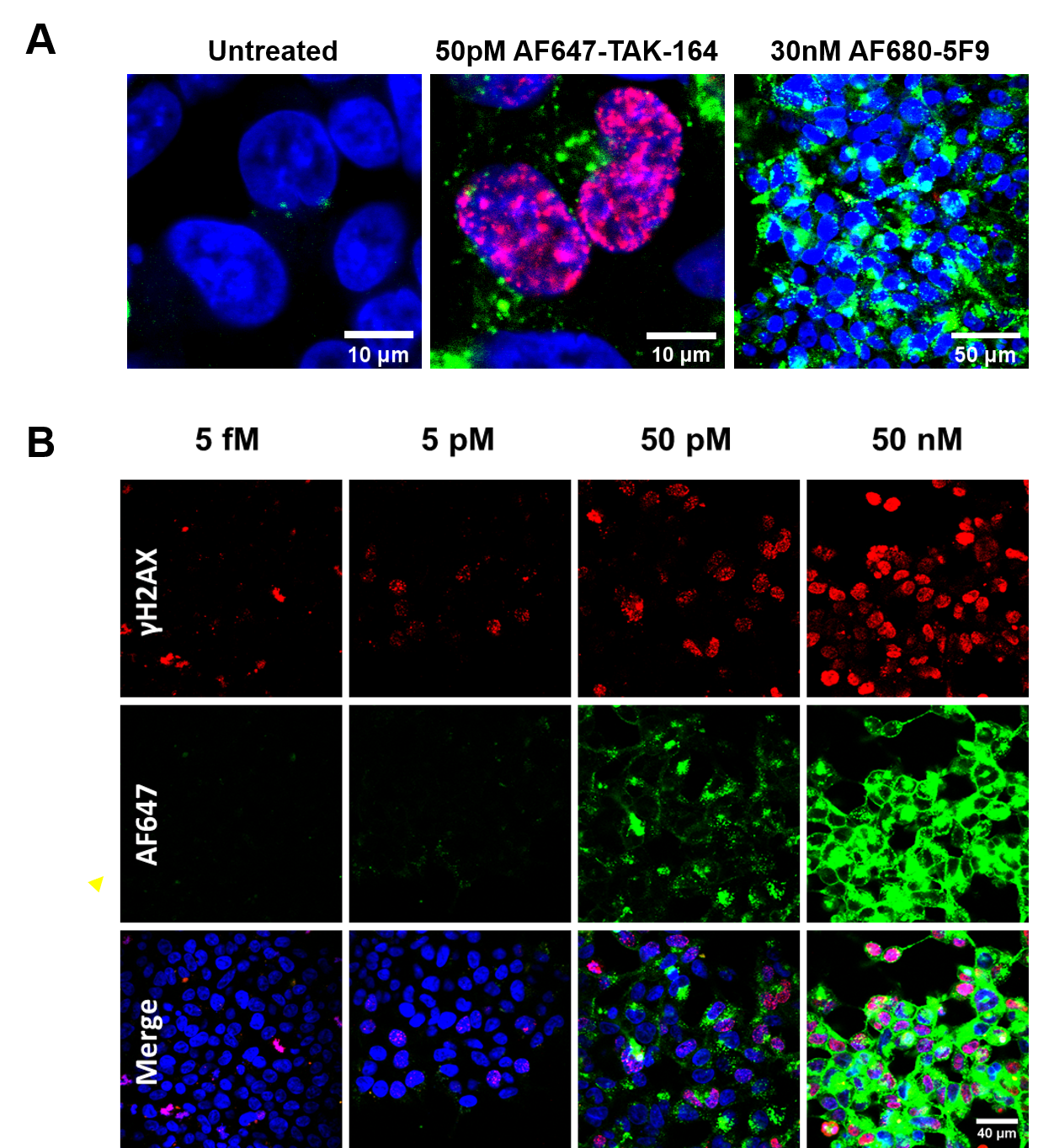


**Supplementary Figure 4. Concentration-dependent phosphorylation of H2A.X.** **(A)** DNA-alkylating DGN549 payload on TAK-164 induces double-stranded DNA breaks (DSBs) that results in phosphorylation of Ser139 residue of H2A.X histone protein (γH2A.X), evidenced by detection of punctate signal in the nucleus at 54 hours (60X high magnification image for higher resolution), but not observed in untreated (60X high magnification) or 5F9 antibody treated (20X low magnification image) cells with immunofluorescence staining. **(D)** *In vitro* staining of monolayer HEK293-GCC cells treated with varying concentrations of fluorescent TAK-164 for 54 hours shows dose-dependent γH2A.X pharmacodynamic response (*green = TAK-164, red = γH2A.X, blue = Hoechst 33342/nucleus*). *Yellow arrowheads indicate mitotic cells showing high γH2A.X signal.*


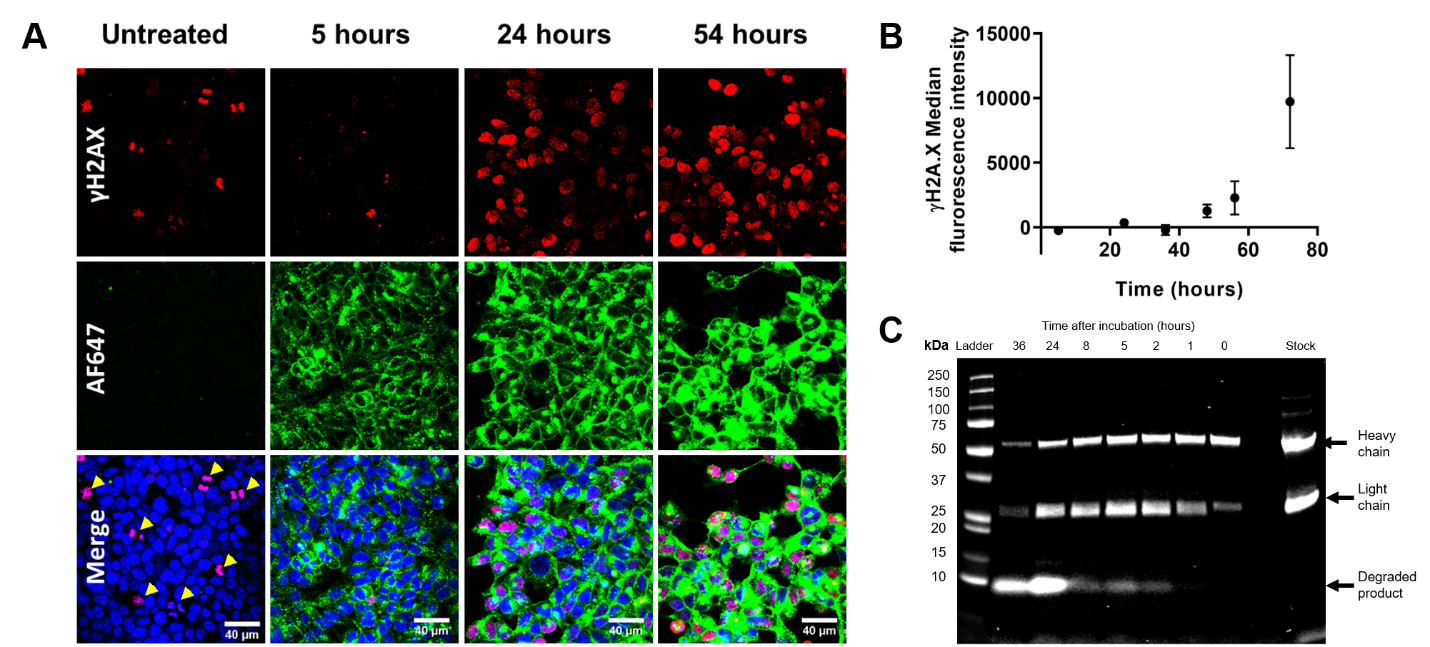


**Supplementary Figure 5. Time-dependent phosphorylation of H2A.X. (A)** Immunohistochemistry of HEK293-GCC cells treated with 50nM AF647-TAK-164 shows stronger γH2A.X signal over time compared to untreated cells, with 5 hours cells showing few, small γH2A.X foci, 24 hours cells showing larger, frequent γH2A.X foci, and 54 hours cells showing more widespread/pan-nuclear staining. γH2A.X staining in untreated group is observed only in cells undergoing mitosis (*yellow arrowheads*), similar to previous literature observations(12). **(B)** Flow cytometry analysis provides quantitative confirmation of temporally increasing γH2A.X signal. **(C)** Reducing SDS-PAGE of lysates of HEK293-GCC cells treated with TAK-164 showing temporal changes in relative quantities of intact ADC (heavy chain, light chain) and degradation products. Appearance of γH2A.X signal in HEK293-GCC cells 24 hours post-incubation with TAK-164 is consistent with an intracellular degradation half-life of ~ 24 hours.


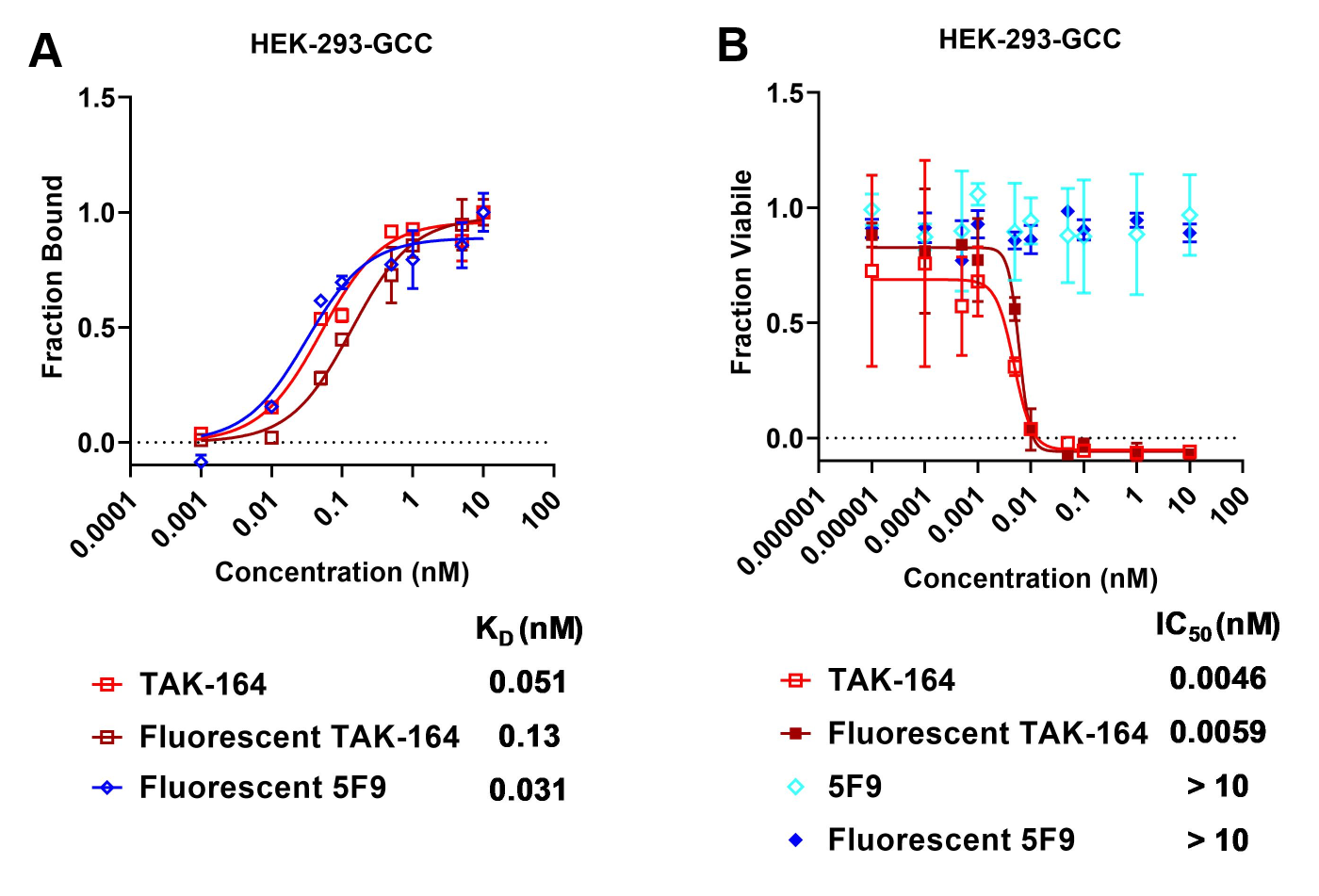


**Supplementary Figure 6. TAK-164 binding affinity and toxicity. (A)** TAK-164 and 5F9 (parent antibody without DGN549 payload) exhibit strong binding affinity (sub-picomolar) to GCC, as determined by flow cytometric cell-based binding affinity protocol described previously(13). **(B)** Cell viability assay shows both fluorescent and non-fluorescent TAK-164 to be similarly ultra-potent (IC_50_ ~ 5pM), while the 5F9 antibody alone did not show any toxicity.


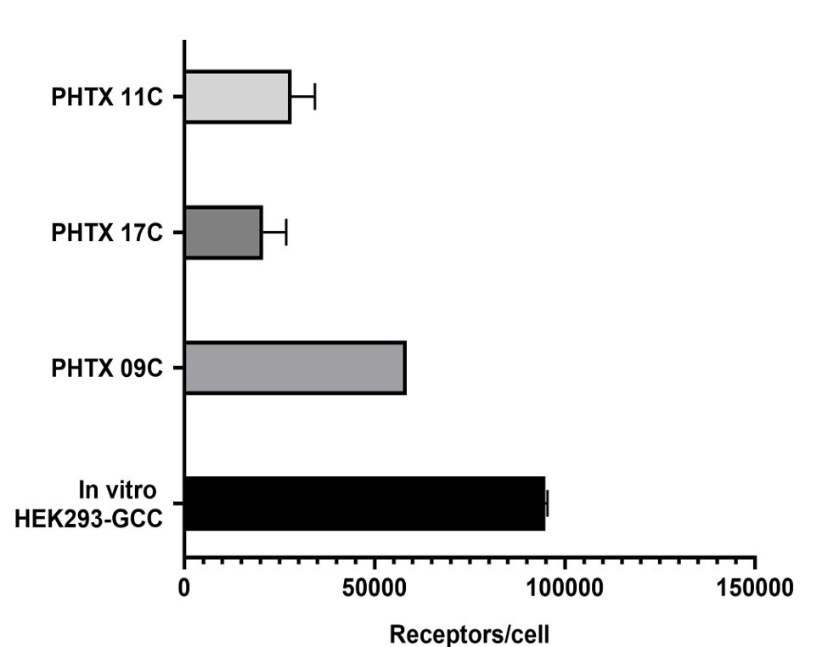


**Supplementary Figure 7. Receptor expression quantification.**  For primary human tumor xenograft (PHTX) models, tumors were harvested from mice, digested into single cell suspensions using a human tumor dissociation kit (Miltenyi Biotec, Germany), and for HEK293-GCC, in vitro cultured cells were used. All the single cell suspensions, along with human IgG calibration beads (Bangs Lab, Fishers, IN) were incubated with 100nM of AF647-TAK-164 on ice for 30 minutes, washed with 1X PBS (Invitrogen, Carlsbad, CA) and analyze via flow cytometry. Calibration beads were used to generate a calibration curve, and the median fluorescence signal from each cell suspension was compared to the calibration curve to quantify the mean surface receptor expression per cell.


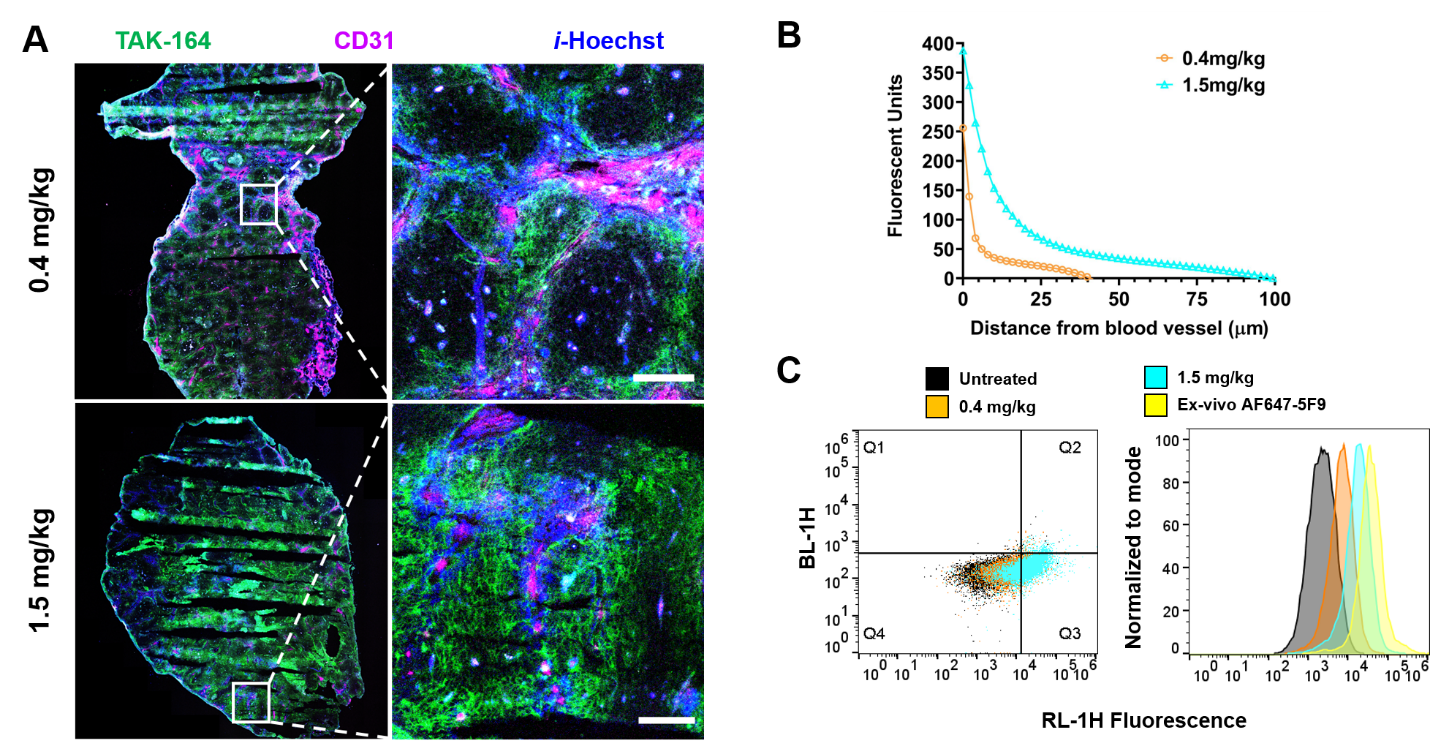


**Supplementary Figure 8. Dose-dependent TAK-164 tumor penetration in PHTX 11C. (A)** Immunohistochemistry of PHTX 11C tumors treated with 0.4mg/kg or 1.5mg/kg AF647-TAK-164 (*green*) qualitatively shows dose-dependent increase in tumor penetration of the ADC. *Magenta = CD31 (all blood vessels), blue = injected Hoechst 33342 (functional blood vessels)*. **(B)** Euclidean distant mapping of full tumor histology scan provides semi-quantitative evidence of improved penetration of 1.5mg/kg TAK-164 (*cyan*) compared to 0.4mg/kg (*orange*). **(C)** Flow cytometry analysis of treated tumors digested to single cell suspensions shows quantitatively that 1.5mg/kg (*cyan*) TAK-164 is closer to a saturating dose compared to 0.4m/kg (*orange*) when compared signal from to digested cells treated ex-vivo with AF647-5F9 (*yellow*).


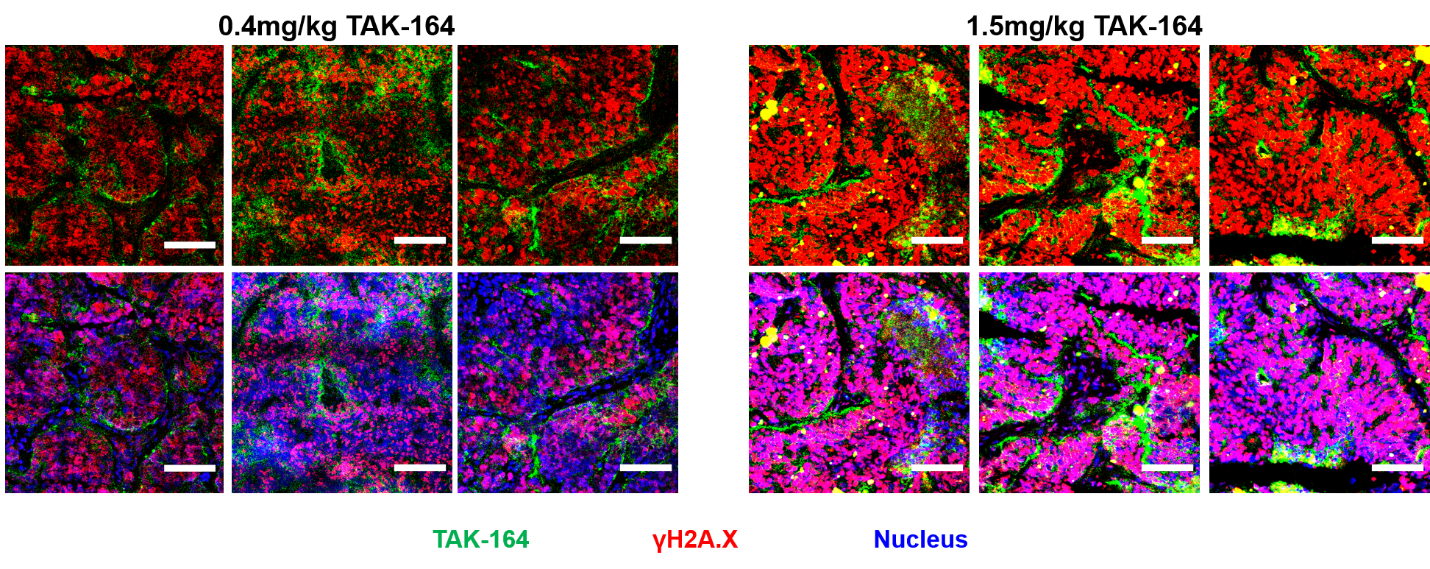


**Supplementary Figure 9. Dose-dependent phosphorylation of H2A.X in PHTX 11C tumors.** Additional immunohistochemistry of PHTX 11C tumors treated with 0.4mg/kg or 1.5mg/kg AF647-TAK-164 (*green*) qualitatively shows dose-dependent increase in γH2A.X signal (*red*), which overlays with nuclear signal from ex-vivo Hoechst 33342 (*blue*). Scalebar is 100µm.


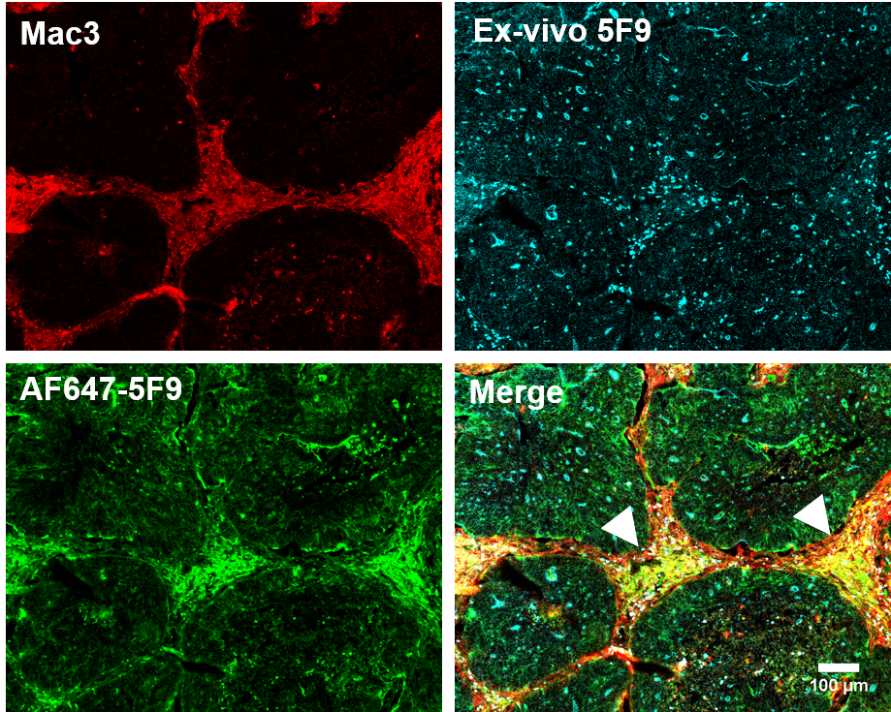


**Supplementary Figure 10. Tumor-associated macrophage (TAM) uptake of 5F9 in PHTX 11C tumors.** Immunohistochemistry of PHTX11C tumors treated with 0.75mg/kg AF647-5F9 (*green*) showed substantial ADC uptake in TAM-rich regions (*red*), indicated by the whit arrowheads. Ex-vivo labeling of tumor sections with AF750-5F9 (*cyan*) showed additional binding in macrophage-rich regions, indicating specific uptake in TAMs rather than non-specific phagocytosis.


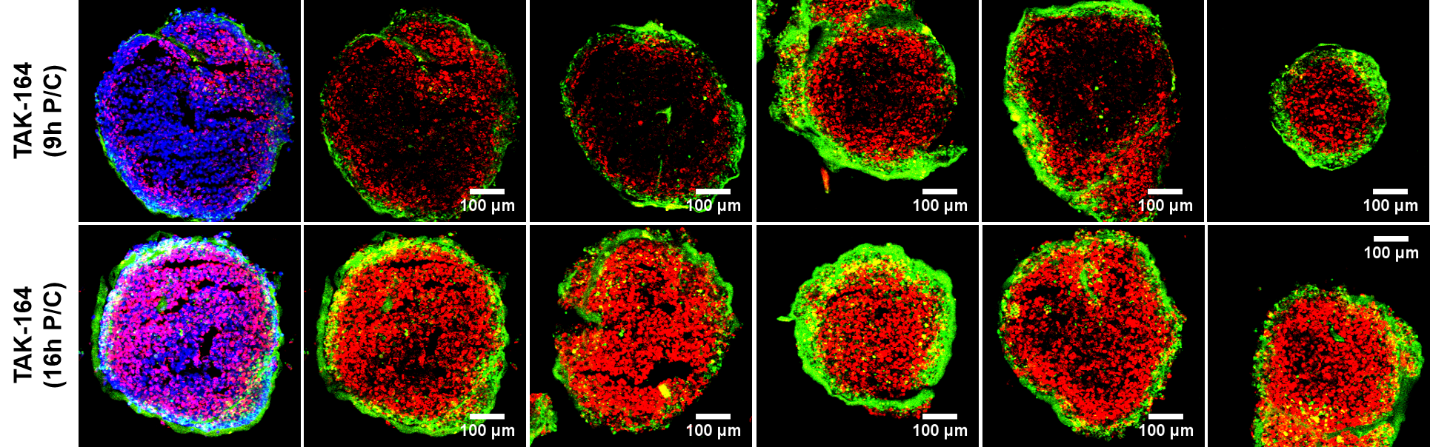


**Supplementary Figure 11. Tunable bystander penetration of DGN549 in tumor spheroids.** Additional immunohistochemistry of HEK293-GCC spheroids pulse-chased with TAK-164 (*green*) showing higher penetration of DGN549 beyond the ADC layer, observed via greater γH2A.X signal (*red*) which overlays with nuclear signal from ex-vivo Hoechst 33342 (*blue*).

**
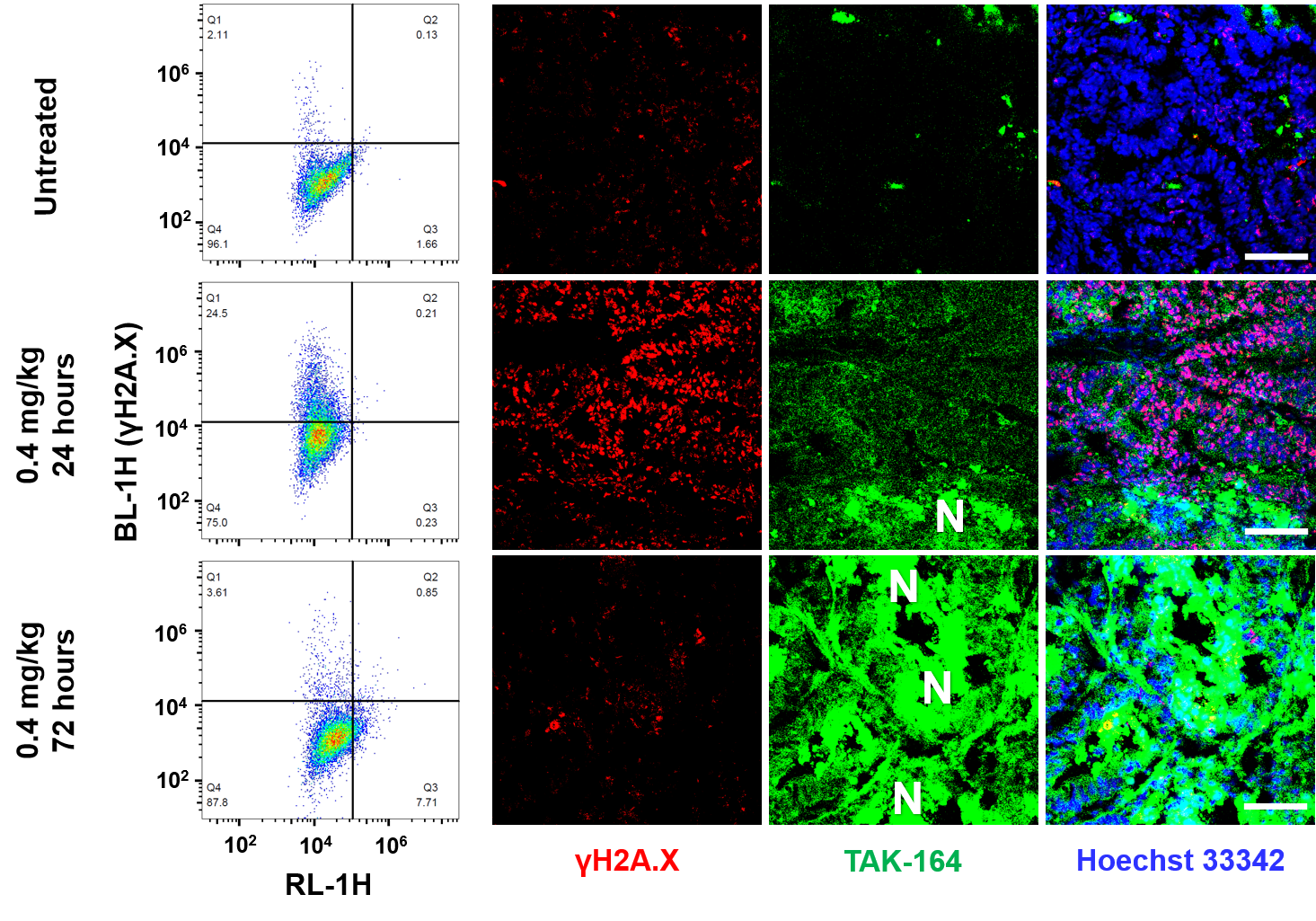
Supplementary Figure 12. Transient nature of γH2A.X in vivo.** PHTX 09C tumors treated with TAK-164 (*green*) showed strong γH2A.X signal (*red*) 24 hours post tail-vein injection, which largely disappeared by 72 hours post tail-vein injection.

**References**

1. Khera E, Cilliers C, Bhatnagar S, Thurber GM. Computational transport analysis of antibody-drug conjugate bystander effects and payload tumoral distribution: implications for therapy. Molecular Systems Design & Engineering **2018**;3:73-88

2. Li C, Dong L, Kamali A, Sugimoto H, Abdul-Hadi K, Chen S*, et al.* An LC/MS based method to quantify DNA adduct in tumor and organ tissues. Anal Biochem **2019**;568:1-6

3. Singh R, Reid EE, Harris L, Salomon PL, Miller ML, Chari RVJ*, et al.* Antibody-Drug Conjugates with Indolinobenzodiazepine Dimer Payloads: DNA-Binding Mechanism of Indolinobenzodiazepine Dimer Catabolites in Target Cancer Cells. Mol Pharm **2020**;17:50-8

4. Thurber GM, Reiner T, Yang KS, Kohler RH, Weissleder R. Effect of small-molecule modification on single-cell pharmacokinetics of PARP inhibitors. Mol Cancer Ther **2014**;13:986-95

5. Bai C, Reid EE, Wilhelm A, Shizuka M, Maloney EK, Laleau R*, et al.* Site-Specific Conjugation of the Indolinobenzodiazepine DGN549 to Antibodies Affords Antibody-Drug Conjugates with an Improved Therapeutic Index as Compared with Lysine Conjugation. Bioconjug Chem **2020**;31:93-103

6. Miller ML, Shizuka M, Wilhelm A, Salomon P, Reid EE, Lanieri L*, et al.* A DNA-Interacting Payload Designed to Eliminate Cross-Linking Improves the Therapeutic Index of Antibody-Drug Conjugates (ADCs). Mol Cancer Ther **2018**;17:650-60

7. Bhatnagar S, Deschenes E, Liao J, Cilliers C, Thurber GM. Multichannel imaging to quantify four classes of pharmacokinetic distribution in tumors. J Pharm Sci **2014**;103:3276-86

8. Bolleddula J, Shadid M, Shah A, Kamali A, Smith M, Abu-Yousif A*, et al.* In vitro and in vivo catabolism of TAK-164, a gcc-targeted antibody-drug conjugate. Drug Metabolism and Pharmacokinetics **2019**;34:S20-S1

9. Thurber GM, Weissleder R. A Systems Approach for Tumor Pharmacokinetics. PLoS One **2011**;6:e24696

10. Thurber GM, Wittrup KD. Quantitative spatiotemporal analysis of antibody fragment diffusion and endocytic consumption in tumor spheroids. Cancer Res **2008**;68:3334-41

11. Pruijn FB, Patel K, Hay MP, Wilson WR, Hicks KO. Prediction of Tumour Tissue Diffusion Coefficients of Hypoxia-Activated Prodrugs from Physicochemical Parameters. Aust J Chem **2008**;61:687-93

12. Tu WZ, Li B, Huang B, Wang Y, Liu XD, Guan H*, et al.* gammaH2AX foci formation in the absence of DNA damage: mitotic H2AX phosphorylation is mediated by the DNA-PKcs/CHK2 pathway. FEBS Lett **2013**;587:3437-43

13. Cilliers C, Nessler I, Christodolu N, Thurber GM. Tracking Antibody Distribution with Near-Infrared Fluorescent Dyes: Impact of Dye Structure and Degree of Labeling on Plasma Clearance. Mol Pharm **2017**;14:1623-33
